# Inhibitory prodrug mechanism for cysteine cathepsin-targeted self-controlled drug release

**DOI:** 10.1080/14756366.2022.2122961

**Published:** 2022-09-19

**Authors:** Floris J. van Dalen, Martijn Verdoes

**Affiliations:** Department of Tumour Immunology and the Institute for Chemical Immunology, Radboud Institute for Molecular Life Sciences, Radboud University Medical Centre, Nijmegen, Netherlands

**Keywords:** Inhibitory prodrug, selective drug delivery, cysteine cathepsins, tumour-associated macrophages, tumour microenvironment

## Abstract

Tumour-associated macrophages (TAMs) support tumour development and have emerged as important regulators of therapeutic response to cytostatic agents. To target TAMs, we have developed a novel drug delivery approach which induces drug release as it inhibits cysteine cathepsin activity. This inhibitory prodrug (IPD) approach establishes a self-regulated system where drug release stops after all cysteine cathepsins are inhibited. This could improve the therapeutic window for drugs with severe side effects. We demonstrate and characterise this self-regulation concept with a fluorogenic IPD model. Next, we applied this IPD strategy to deliver cytotoxic drugs, as doxorubicin and monomethyl auristatin E, which are efficiently released and dose-dependently eliminate RAW264.7 macrophages. Lastly, by exploiting the increased cathepsin activity in TAM-like M2-polarised primary macrophages, we show that IPD-Dox selectively eliminates M2 over M1 macrophages. This demonstrates the potential of our IPD strategy for selective drug delivery and modulation of the tumour microenvironment.

## Graphical Abstract

**Figure:**
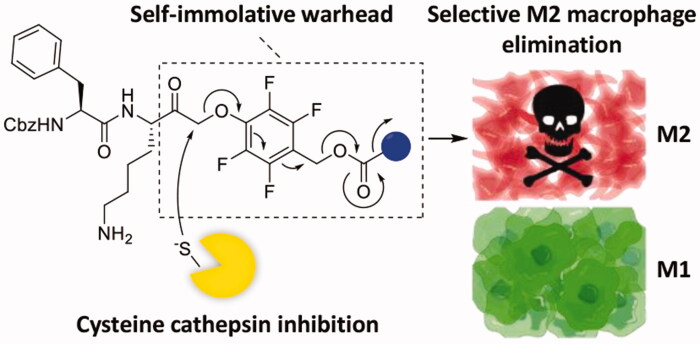

## Introduction

Chemotherapy remains the first line of defence against cancer. However, most anticancer drugs suffer from dose-limiting adverse effects. For instance, the use of doxorubicin is restricted by dose-accumulating cardiotoxicity^1^^,^^2^. Selective delivery of cytotoxic drugs is an attractive strategy to improve the therapeutic window. Substrate prodrugs employ tumour-overexpressed enzymes to locally trigger drug activation. Cysteine cathepsins (cCTSs) are highly upregulated in cancer and support tumour development in all stages of disease^3^. cCTSs cleave the extracellular matrix and cell adhesion molecules paving the way for invading tumour cells^3–7^. Moreover, they are involved in activating growth factors and angiogenesis^3^^,^^8^^,^^9^. In the tumour microenvironment (TME), cCTS activity is predominantly localised in tumour-associated macrophages (TAMs) ^10^^,^^11^. TAMs can make up a significant portion of the tumour mass (up to 30%) and support tumour development into malignancy^12^^,^^13^. They display an M2-like phenotype, hallmarked by immunosuppressive factors (e.g., interleukin 10, programmed death ligand 1, transforming growth factor beta), increased secretion of angiogenic molecules (e.g., adrenomedullin and vascular epithelial growth factors) and an increase in matrix metalloprotease- and cCTS-activity^14^. TAMs have emerged as important regulators of therapeutic response to cytostatic agents and present an immunosuppressive barrier for effector functions of T lymphocytes and NK cells^12–14^. Therefore, cCTS activity in TAMs presents an attractive target for cancer treatment.

**Figure 1.**
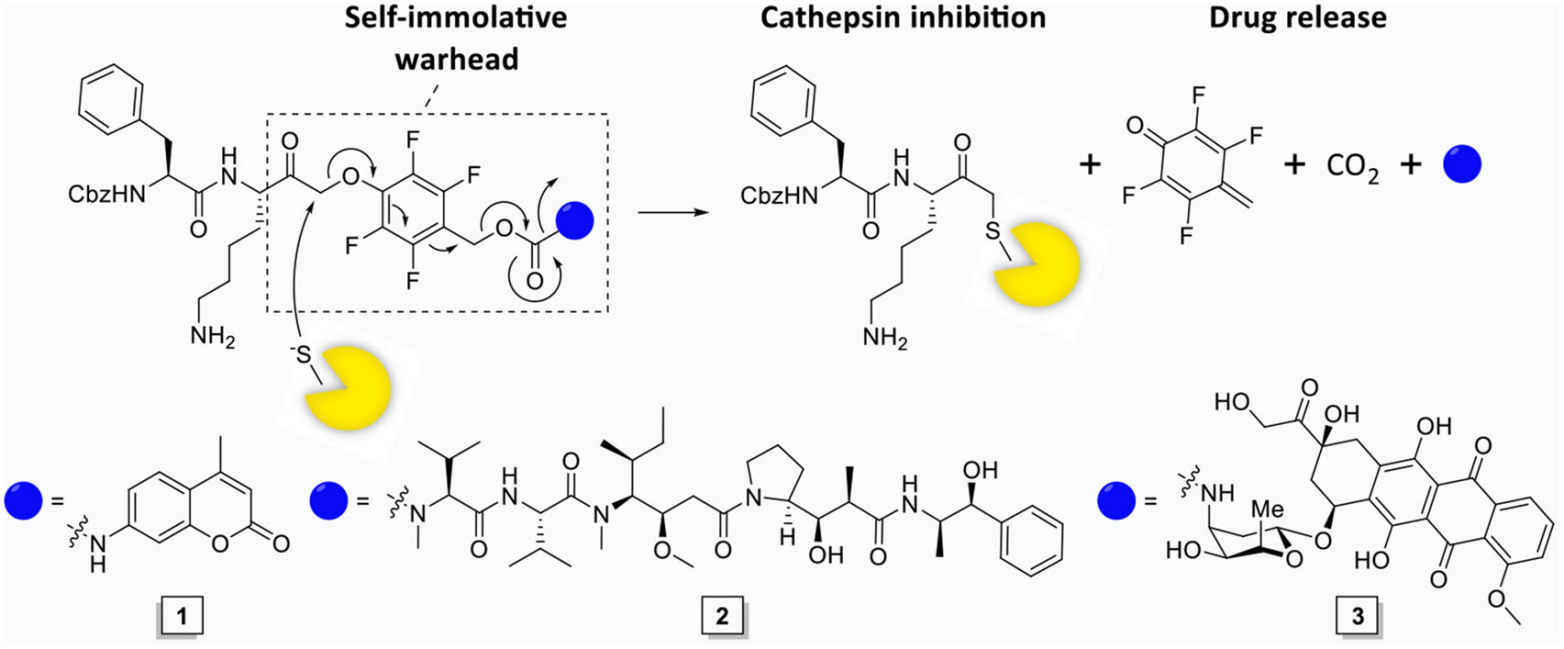
The mechanism of action of the self-immolative warhead and structures of IPD-AMC (**1**), IPD-MMAE (**2**), and IPD-Dox (**3**). In short, nucleophilic attack on the phenoxymethyl ketone results in cathepsin inhibition followed by self-immolation and payload release.

Proteases in cancer have become an important field of research over the past decades and have become an established therapeutic target for protease inhibitors and protease-activated prodrugs^15^^,^^16^. cCTS activity in TAMs can be exploited as diagnostic marker (e.g., in fluorescence guided surgery)^17^, as therapeutic target for cCTS inhibitors^18^ or to facilitate local drug activation (e.g., in prodrugs and antibody drug conjugates)^19–22^. However, current prodrug strategies allow cCTS-activity to continue along with the associated tumour-promoting processes. While pharmalogical inhibition of cCTSs has been demonstrated to reduce tumour malignancy in preclinical models and exhibits synergistic effects with cytostatic agents such as cyclophosphamide and doxorubicin^18^^,^^23^. Therefore, we designed a single molecule prodrug approach that simultaneously inhibits cCTSs as it induces drug release, through the development of a self-immolative warhead (Figure 1). This design, dubbed inhibitory prodrug (IPD), establishes a self-regulated system where drug release stops after all cCTSs are inhibited. This will potentially broaden the therapeutic window for drugs with severe side effects. Furthermore, this form of drug delivery could intrinsically synergise cCTS inhibition and cytotoxic agents by targeting TAMs in two distinct manners. That is, invasion, metastasis and angiogenesis are reduced by cCTS inhibition, after which the cytotoxic agent can eliminate immunosuppressive TAMs and could potentially kill adjacent tumour cells through the bystander effect.

## Material and methods

### IPD synthesis

**Scheme 1.**
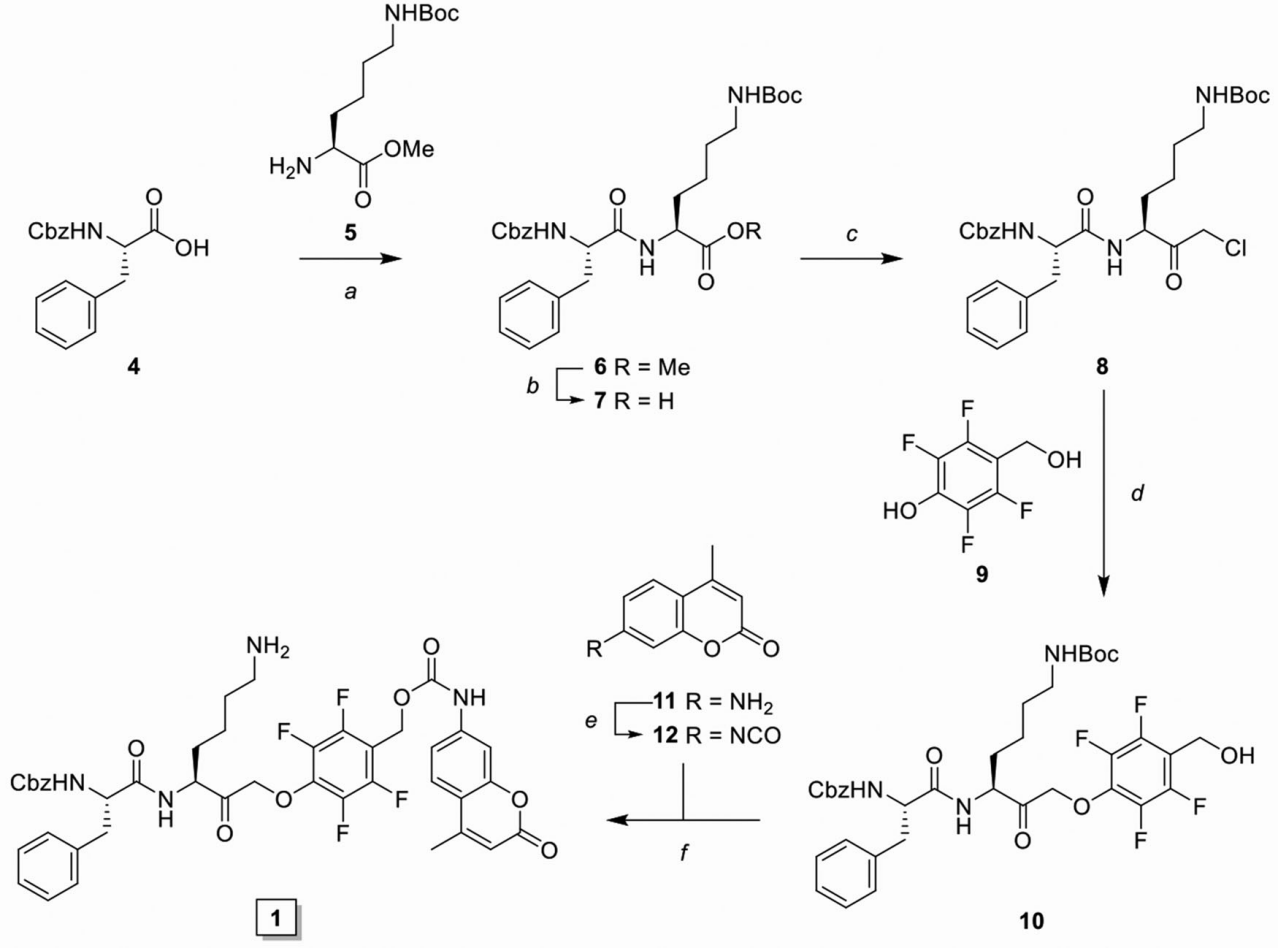
Synthesis of inhibitory prodrug model **1** (IPD-AMC). Reagents and conditions: a) HBTU, DiPEA, DMF, r.t., quant.; b) LiOH·H_2_O, THF/MeOH/H_2_O, r.t., 1 h, quant.; c) *i*. *N-*methyl morpholine, isobutyl chloroformate, THF, -15 °C, 30 min, *ii*. CH_2_N_2_ in Et_2_O, THF, -15 °C, 3 h, *iii*. HCl/AcOH, -15 °C, 10 min, 85% over 3 steps; d) **9**, KF, DMF, 70 °C, 16 h, 80%; e) 20% phosgene in toluene, reflux, 16 h; f) *i*. isocyanate 12, dibutyltin diluarate (5 mol%), THF, r.t., 16 h., *ii*. TFA/DCM, r.t., 30 min, 25% over 2 steps.

**Scheme 2.**
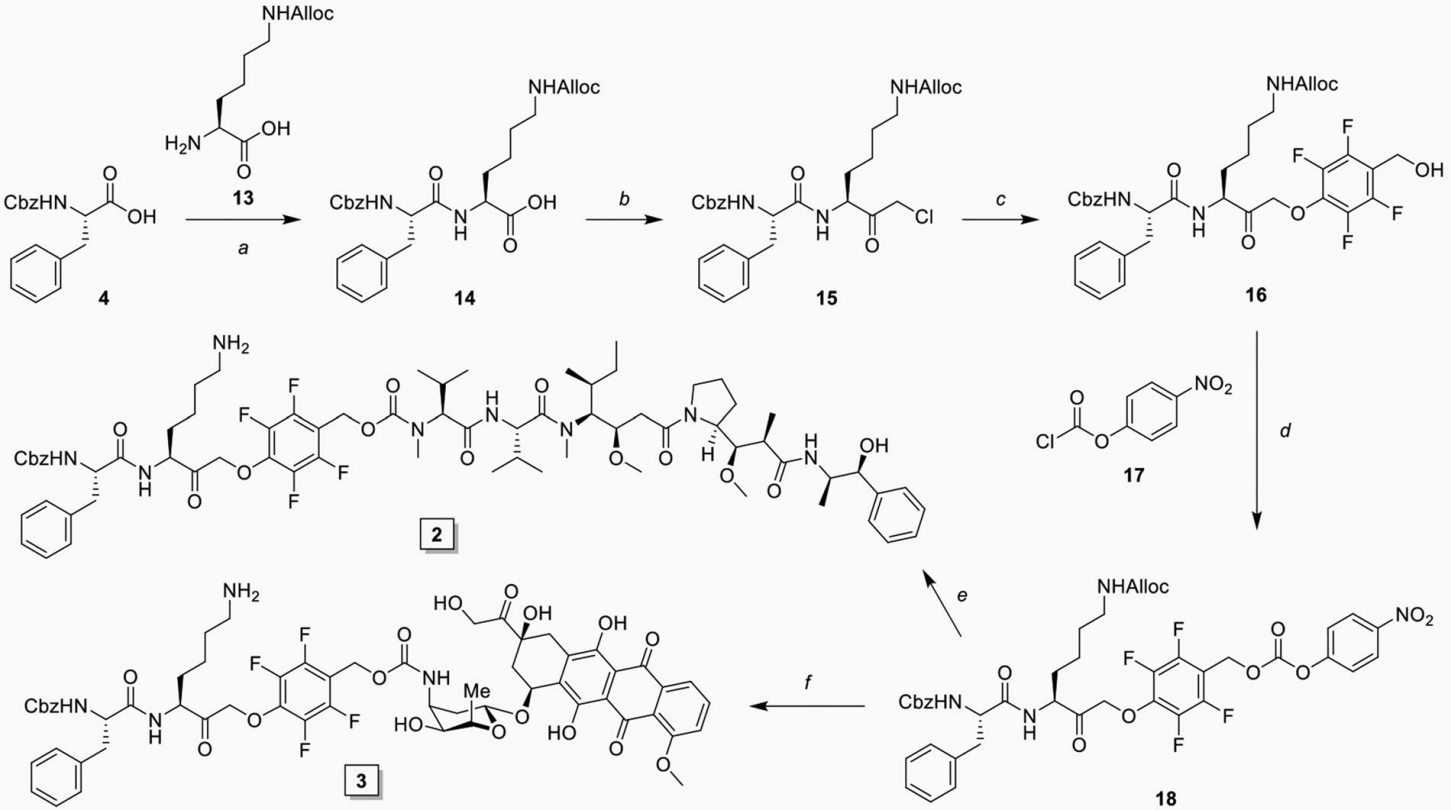
Synthesis of inhibitory prodrug **2** (IPD-MMAE) and **3** (IPD-Dox). Reagents and conditions: a) *i.* TSTU, DiPEA, DMF, 0 °C, 1 h; *ii.* 11, r.t., 16 h, 92%; b) *i*. *N-*methyl morpholine, isobutyl chloroformate, THF, -15 °C, 15 min, *ii*. CH_2_N_2_ in Et_2_O, THF, -15 °C, 3 h, *iii*. HCl/AcOH, -15 °C, 10 min, 80% over 3 steps; c) **9**, KF, DMF, 60 °C, 3 h, 79%; d) 17, DMAP, DCM, -20 °C to r.t., 4 h, 99%; e) *i.* MMAE, HOBt, Pyridine, DMF, r.t., 48 h, *ii.* Pd(PPh_3_)_4_, DMBA, DCM, 15 min, r.t., 71% over 2 steps; f) *i.* Doxorubicin, DiPEA, DMF, r.t., 48 h, *ii.* Pd(PPh_3_)_4_, DMBA, DCM, 15 min, r.t., 59% over 2 steps.

Compounds **1 − 3** were synthesised as depicted in Schemes 1 and 2. The synthesis of IPD-Ctrl **23** is described in Scheme S1. Detailed experimental procedures and analytical data can be found in the supplemental information.

### Cell culture

RAW264.7 cells were maintained in DMEM (Gibco^®^) containing high glucose, stable glutamine (GlutaMAX™), sodium pyruvate and phenol red, which was supplemented with 10% foetal calf serum (FCS, Bio-Greiner One) and antibiotics (100 units/ml penicillin, 100 µg/ml streptomycin, and 250 ng/ml amphotericin B (Gibco^®^)). The cells were cultured in T75 flask (Corning) in a humidified 5% CO_2_-atmosphere at 37 °C and the culture was passaged every 2–3 days. The cells were suspended via cell scraper, followed by centrifugation at 1000x *g* for 5 min at 4 °C. The medium was refreshed and the cells were seeded to the appropriate confluence. Cells were harvested at 80–90% confluence.

### BMDM preparation

Mouse bone marrow was isolated from the femur and tibia from 8–12 weeks old female wild type C57BL/6J mice (Charles River, France). In short, hind legs were dissected and muscle tissue was removed. Bones were cleaned in 70% ethanol for 3 min, washed with PBS and conditioned medium; RPMI1640 (Gibco) containing HEPES and phenol red, which was supplemented with FCS (10%), antibiotics (100 units/ml penicillin, 100 µg/ml streptomycin, and 250 ng/ml amphotericin B (Gibco)), 2 mM ultraglutamine (Lonza) and 50 µM β-mercaptoethanol (Sigma Aldrich, freshly added from 50 mM aliquots prepared under oxygen-poor conditions and stored at −20 °C). Bones were cut with a scalpel, marrow was flushed out with 5–10 ml complete medium and filtered over 100 µm mesh strainer (Corning). The cells were centrifuged at 1000x *g* for 5 min at 4 °C and the red blood cells were lysed with ACK lysis buffer (150 mM NH_4_Cl, 10 mM KHCO_3_, 0.1 mM disodium EDTA) 30 s on ice. 50 ml PBS was added and cells were centrifuged at 1000x *g* for 5 min at 4 °C. Cells (2·10^6^) were seeded in a non-culture treated 10 cm PS petridish (Falcon) and bone marrow-derived macrophages were obtained by differentiation under influence of recombinant mouse 20 ng/ml M-CSF (all mouse cytokines were sourced from Peprotech) in 10 ml complete medium. The cells were cultured in a humidified 5% CO_2_-atmosphere at 37 °C and the medium was supplemented on day 3 with 5 ml complete medium containing 20 ng/ml M-CSF. On day 5, the medium was refreshed and cells were activated to form M0 (20 ng/ml M-CSF), M1 (20 ng/ml GM-CSF, 50 ng/ml IFNγ and 100 ng/ml LPS) and M2 (20 ng/ml M-CSF and 10 ng/ml IL-4) populations. At day 6 the floating population (<5% of cells) was removed by washing with PBS and the adherent population was harvested via cell scraper.

### Lysate preparation

Cellular lysate was prepared from RAW264.7 culture harvested at 70–90% confluence. The cells were suspended, per described method, followed by centrifugation at 1000x g for 5 min at 4 °C. The supernatant was removed and the pellet was suspended in 10 µl per 1·10^6^ cells citrate buffer (50 mM citric acid pH 5.5, 5 mM DDT, 0.5% CHAPS, and 0.1% Triton X-100). The mixture was put on ice for 15 min, sonicated 3 × 5 s on ice, followed by centrifugation at 21 130x g for 15 min at 4 °C. The cleared lysate was transferred into pre-cooled Eppendorf’s (0.5 ml aliquots) and stored at −20 °C.

### CABPP in whole cells or lysate

RAW264.7 macrophages or BMDMs (some 2·10^5^ cells in 100 µl conditioned medium) were incubated with the indicated concentration of inhibitor (200x in DMSO) for 1 h at 37 °C, followed by labelling with 1 µM BMV109 (200x in DMSO) for 1 h at 37 °C. The cells were centrifuged at 10 000x *g* for 1 min at r.t., the supernatant was removed, and the cells were taken up in 9 µl hypotonic lysis buffer (50 mM PIPES pH 7.4, 10 mM KCl, 5 mM MgCl, 4 mM DTT, 2 mM EDTA, and 1% NP40). The lysate was incubated on ice for 5 min, followed by centrifugation at 21 130x *g* for 15 min at 4 °C. The cleared lysate was diluted with 3 µl Laemmli’s 4x sample buffer (40% glycerol, Tris/HCl (0.2 M, pH 6.8), 8% SDS, 10% BME, and 0.04% bromophenol blue) and the mixture was denatured over 5 min at 95 °C. The samples were spun down and separated by SDS PAGE (15%, 15 min at 80 V, 1.5–2 h at 120 V). The gel was analysed by in-gel fluorescence scanning on a Typhoon Trio flat-bed laser scanner (GE Healthcare) and equal protein loading was confirmed by staining with Coomassie^®^ Brilliant Blue R-250 (Schmidt GmbH).

RAW264.7 lysate (10 µl, in citrate buffer pH 5.5) was incubated with the indicated concentration of inhibitor (20x in citrate buffer) for 1 h at 37 °C, followed by labelling with 1 µM BMV109 (20x in citrate buffer) for 1 h at 37 °C. The solution was centrifuged (15 min, 21 130x g, 4 °C), transferred to clean Eppendorf’s and 4x sample buffer (3 µl) was added. The samples were denatured over 5 min at 95 °C and separated by SDS PAGE (15%, 15 min at 80 V, 1.5–2 h at 120 V, 4 °C). The gel was imaged on a Typhoon Trio (GE Healthcare), and constant protein loading was confirmed by staining with Coomassie^®^ Brilliant Blue R-250 (Schmidt GmbH).

cABPP-labelling intensities were quantified using Image J software. Data was transferred to Microsoft Excel, corrected for background fluorescence, and scaled to the positive control (DMSO, BMV109) as 100%-activity reference point. The mean, standard deviation (SD), and standard error of the mean (SEM) were calculated and normalised to the corrected positive control. The data was transferred to Graphpad Prism 6.0 and IC50-values were calculated using non-linear regression.

### MTT assay

RAW264.7 cells (some 5·10^3^ cells) or BMDMs (some 5·10^4^ cells) were seeded in a flat-bottom 96-well plate (Bio-Greiner one). The cells were incubated with the indicated concentration of inhibitor or vehicle for 1 h at 37 °C after which the cells were treated with the indicated compounds for 3 days in a humidified 5% CO_2_-atmosphere incubator at 37 °C. The medium was replaced with 60 µl conditioned medium and cells were incubated with 10 μl MTT (3–(4,5-Dimethylthiazol-2-yl)-2,5-Diphenyltetrazolium Bromide) (Sigma-Aldrich) solution (4 mg/ml) for 1 h at 37 °C. The medium was removed and the formed formazan crystals were dissolved in 100 µl acidic lysis buffer (90% isopronanol, 0.1% SDS, 40 mM HCl in water) for 1 h at 37 °C. The OD595 of the dissolved crystals was measured with a BioRAD iMark microplate absorbance reader.

### AMC-release assay

RAW264.7 cell lysate (20 µl, corresponding to some 2·10^6^ cells, pH 5.5) was treated with the indicated concentration of inhibitor (40x in citrate buffer) or vehicle for 1 h at 37 °C, followed by incubation with the indicated concentration of IPD-AMC (40x in citrate buffer) for 1 h at 37 °C. Experiments without pre-treatment were directly incubated with IPD-AMC (40x in citrate buffer) for 1 h at 37 °C. Citrate lysis buffer incubated with IPD-AMC served as internal control. The fluorescent signal (λ_ex_ = 360 ± 5 nm, λ_em_ = 440 ± 5 nm, r.t.) was measured on a LS 55 spectrophotometer (Perkin Elmer).

### Time-dependent labelling in RAW264.7 lysate

RAW264.7 cell lysate (from 2·10^6^ cells) was incubated with IPD-AMC (2.5 µM) for the indicated time periods. A sample (2.5 µl) of the incubated lysate was transferred to BMV109 (2.78 µM, 22.5 µl) to quench the inhibition reaction and label residual cathepsin activity with BMV109. The resulting mixture was incubated for 1 h at 37 °C. The proteome was cleared by centrifugation (15 min, 21 130x *g*, 4 °C), the supernatant was sampled (9 µl), and diluted with 4x sample buffer (3 µl). The proteins were denatured for 5 min at 95 °C and were resolved on SDS PAGE. The labelling intensities were imaged by in-gel fluorescence scanning on a Typhoon Trio (GE Healthcare) and labelling was quantified with Image J Software.

## Results and discussion

### Synthesis and biochemical analysis of a model inhibitory prodrug

**Figure 2.**
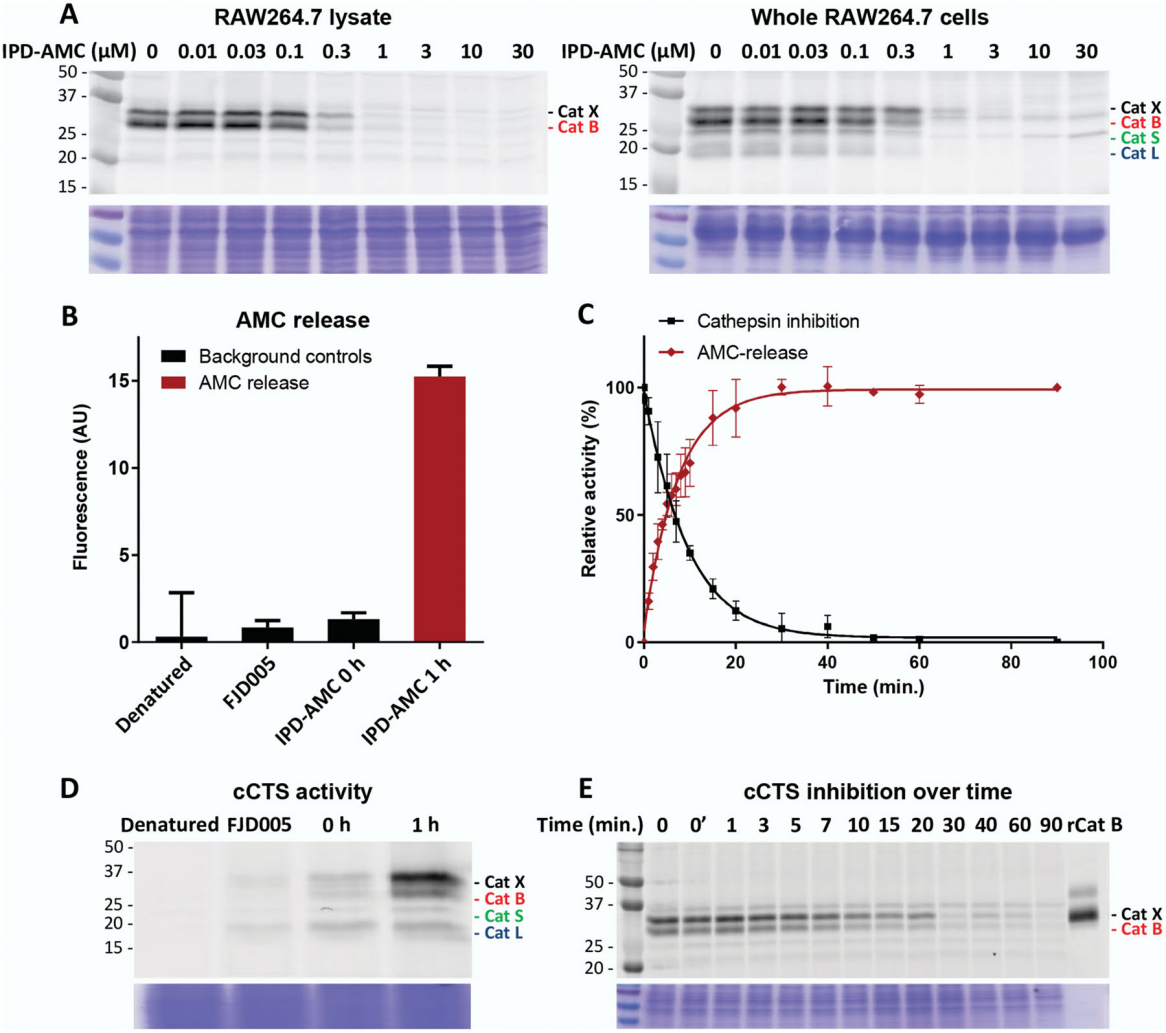
Cathepsin inhibition and AMC release by IPC-AMC. A) RAW264.7 lysate (corresponding to 2·10^5^ cells) or live RAW264.7 cells (2·10^5^ cells) were incubated with indicated concentration of IPD-AMC (1 h, 37 °C) after which residual cathepsin activity was labelled with BMV109 (1 µM, 1 h, 37 °C). Cells were lysed and proteomes were separated by SDS PAGE. Cathepsin labelling was visualised by in-gel fluorescence scanning (*N* = 2, *n* = 2). B) AMC release from IPD-AMC (2.5 µM) in RAW264.7 lysate is blocked after deactivating the lysate by denaturation (5 min, 95 °C) or inhibition of cCTSs by inhibitor FJD005 (10 µM, 5 min, 37 °C) (*n* = 3). C) AMC release correlates with cathepsin inhibition over time as determined by in tandem competitive labelling with BMV109. This indicates immediate AMC release following cathepsin inhibition at the observed time-scale (*n* = 3). D) In tandem (c)ABPP for experiment B: RAW264.7 lysate was either pre-treated by denaturation (95 °C, 5 min) or inhibition with inhibitor FJD005 (10 µM, 37 °C for 5 min), followed by labelling with BMV109. BMV109 in RAW264.7 lysate (at t = 0) serves as background control. E) In tandem cABPP for experiment C: RAW264.7 lysate was treated with IPD-AMC (2.5 µM) for the indicated time points after which reaction was quenched by addition of BMV109 (25 µM, final conc.). The lysate was cleared by centrifugation, the proteome separated by SDS PAGE and fluorescent labelling was visualised by in gel fluorescent scanning. Coomassie staining was used to determine equal protein loading.

As initial proof-of-concept we designed and synthesised a model IPD (**1**, IPD-AMC) containing a latent fluorophore, 7-amino-4-methylcoumarin (AMC) (Figure 1 and Scheme 1). This fluorophore remains quenched until it is released from the IPD, with a quenching efficiency of >99% for intact IPD-AMC (Figure S1A). IPD-AMC was synthesised using a modified procedure of the reported phenoxymethyl ketone (PMK) synthesis^10^. In short, Z-phenylalanine **4** was condensated with protected lysine **5**, followed by saponification with lithium hydroxide to yield dipeptide **7**. The corresponding mixed anhydride was prepared with isobutyl chloroformate and was reacted with diazomethane. The formed α’-diazomethyl ketone was treated with hydrogen chloride in acetic acid yielding chloromethyl ketone (CMK) **8**. The CMK was substituted with phenol **9** to form PMK **10** and the regioselectivity was confirmed with 2 D correlation NMR (supplementary data). AMC was activated with 20% phosgene in toluene to form the isocyanate, which was reacted with PMK **10**. Preparatory HPLC and subsequent lyophilisation yielded the desired IPD-AMC **1**. To examine the inhibitory potency of IPD-AMC **1** we performed a competitive activity-based protein profiling (cABPP) experiment. Intact RAW264.7 macrophages (mouse monocytic leukemic macrophage cell line) or RAW264.7 lysate were treated with a titration of IPD-AMC and the residual cathepsin activity was determined with pan-reactive probe BMV109 (Figure 2(A))^10^. This demonstrated complete cathepsin inhibition at approximately 1 µM IPD-AMC in both lysate and live cells, which is in the same order of magnitude as the pentafluoro-PMK inhibitor FJD005 (Figure S5). This shows that IPD-AMC is efficiently internalised by cells and that attachment of the molecular cargo at the prime site does not interfere with cathepsin binding. To determine whether this inhibition also resulted in release of the fluorogenic cargo, we measured fluorogenic activation of AMC upon exposure of RAW264.7 lysate to 2.5 µM IPD-AMC for 1 h (Figure 2(B)). This produced AMC fluorescence corresponding to some 50 nM (∼15 AU), which was reduced to background levels either by denaturing the proteins in the lysate or by pre-treatment with cathepsin inhibitor FJD005, demonstrating that AMC release is controlled by cathepsin activity (Figure 2(B,D)). Because cargo release proceeds in two steps (nucleophilic displacement of the phenol by the active-site cysteine, followed by self-immolation and AMC release), we investigated the correlation between cCTS inhibition- and AMC fluorescent activation-kinetics with a tandem cABPP and AMC release experiment (Figures 2(C,E)). This demonstrated that AMC activation and cathepsin inhibition both plateau after 30 min, indicating that AMC is released in a concerted action upon target inhibition (at the investigated time-scale). This assures that drug activation will remain localised in cells or environments with high cathepsin activity.

### Synthesis and evaluation of cytotoxic IPDs

**Figure 3.**
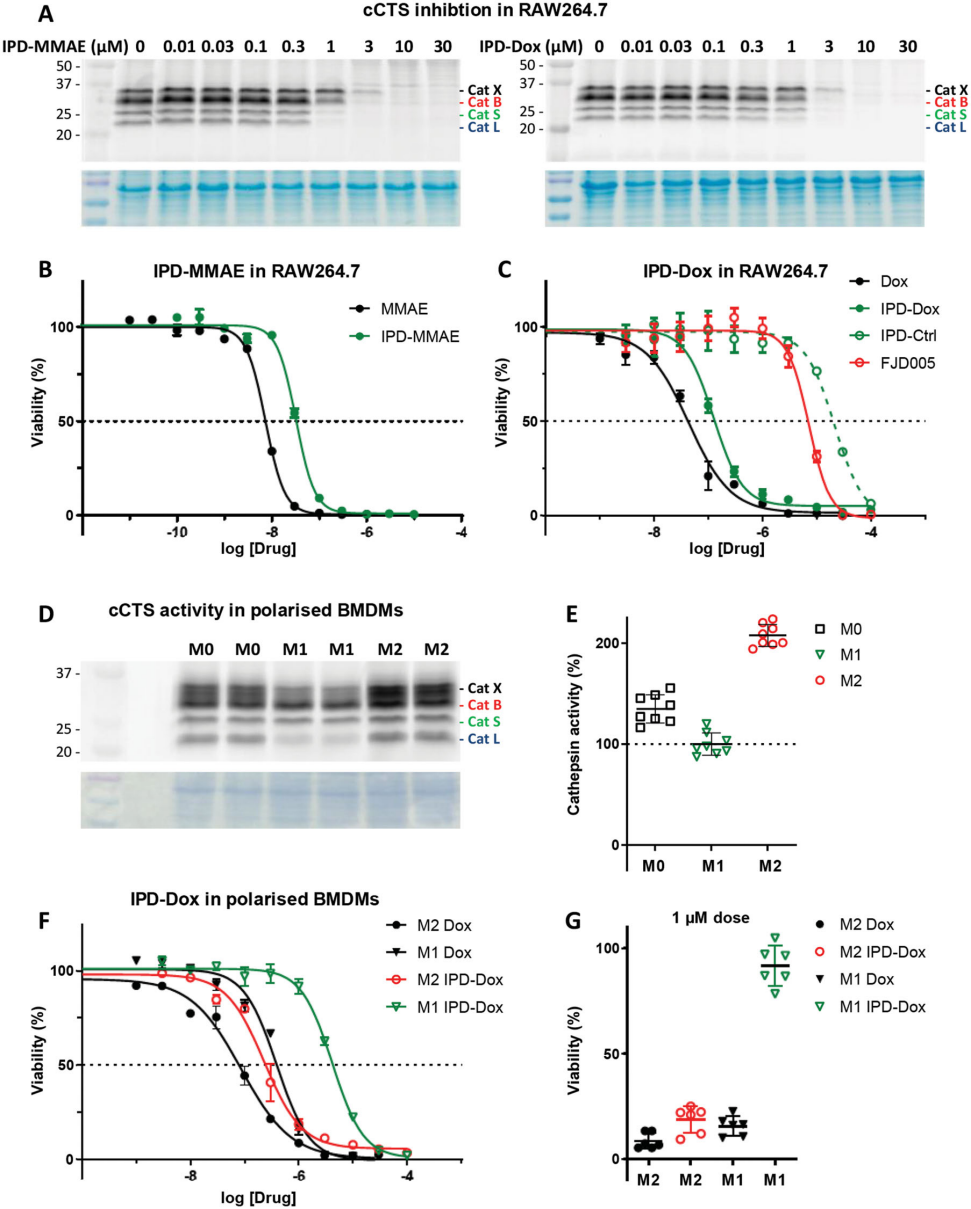
Inhibitory profile and cell killing of cytotoxic IPDs IPD-MMAE and IPD-Dox. A) Live RAW264.7 cells were incubated with indicated concentration of IPD-MMAE and IPD-Dox (1 h, 37 °C), followed by labelling with BMV109 (1 µM, 1 h, 37 °C). Proteomes were separated by SDS PAGE and visualised by in-gel fluorescence scanning (*n* = 2). Coomassie staining was used to determine equal protein loading. B, C) RAW264.7 cells were treated with indicated concentration of cytostatic drug or IPD for 3 days, after which cell viability was assessed by MTT assay (*N* = 3, *n* = 2). D) Bone marrow-derived macrophages were polarised into M0, M1 or M2 subsets and relative cathepsin activity was determined by labelling with BMV109 (1 µM, 1 h, 37 °C) (*n* = 8). Coomassie staining was used to determine equal protein loading. E) Quantification of experiment D. F) M1 and M2 BMDMs were treated with indicated concentrations of doxorubicin or IPD-Dox for 3 days, after which cell viability was assessed by MTT assay. M2 macrophages display higher sensitivity towards doxorubicin (some 4-fold) com-pared to M1 macrophages and IPD-mediated delivery increases this selectivity (some 18-fold) (*n* = 6). G) M2-selective killing by IPD-Dox can be achieved at 1 µM concentration *in vitro* (*n* = 6).

An important consequence of our IPD design is the release of equimolar amounts of drug cargo relative to the concentration of active cCTSs. Therefore, the inherent toxicity of the payload needs to be carefully considered to obtain the required IPD potency and selectivity. We chose two cytotoxic drugs with different toxicities: monomethyl auristatin E (MMAE) with 1–10 nanomolar toxicity and doxorubicin (Dox) in the 10–100 nanomolar range. Next, we synthesised the corresponding IPD-MMAE (**2**) and IPD-Dox (**3**) (Figure 1 and Scheme 2). Synthesis followed the general scheme used for IPD-AMC, however required two adjustments. The Boc-protection was replaced with Alloc due to acid-sensitivity of Dox. Secondly, intermediate **16** was activated with para-nitrophenol chloroformate to form activated intermediate **18** to facilitate ligation with the selected cytostatic drugs. Condensation with Dox or MMAE followed by deprotection with palladium(tetrakistriphenyl)phosphine and subsequent HPLC purification, yielded the desired IPD-Dox **2** and IPD-MMAE **3**, respectively. The inhibitory potency was determined by cABPP with BMV109, which displayed similar potency as IPD-AMC in live RAW264.7 macrophages (Figure 3(A)). To measure IPD toxicity we treated RAW264.7 cells with the free cytostatic agents or corresponding IPDs for three days and assayed cell viability with an MTT assay (Figure 3(B,C)). This resulted in a half-maximal effective concentration (EC50) of 7.3 nM for MMAE and 43 nM for Dox, where the IPDs displayed efficient activation for both cytotoxic payloads, namely 32 nM and 125 nM for IPD-MMAE and IPD-Dox, respectively (Figure 3(B,C) and Table S1). This 3-fold reduction in toxicity is probably a result of the inhibitory nature of the delivery system, where payload release is directly coupled to the concentration of active cathepsins. To determine whether toxicity is dependent on drug activation, we synthesised a control IPD (IPD-Ctrl), containing a non-immolative PMK warhead to maintain cCTS inhibition but prevent the release of active Dox (Figure S2 and Scheme S1). This indeed increased the EC50 in RAW264.7 macrophages two orders of magnitude (>10 µM, Figure 3(C)). This is at a similar level as the toxicity of FJD005 observed at very high concentrations (>10 µM) (Figure 3(C)). This sensitivity for the PMK warhead in macrophages might be the result of NPLR3 inflammasome activation through off-target inhibition of GAPDH or α-enolase as described by Sanman et al.^24^. Altogether, this confirms the crucial importance of Dox release from IPD-Dox, via the self-immolative mechanism initiated by cCTS inhibition, to mediate the cytotoxic action of Dox.

Alternatively, we attempted to determine the cathepsin dependency of IPD toxicity by inhibiting cCTSs prior to treatment, by preincubation with FJD005. However, this preinhibition could not rescue cell viability (Figure S3). Instead, we noticed sensitisation to further treatment, indicating possible synergy between the PMK warhead and cytotoxic agents. To avoid these potential off-target effects we next tried preinhibition with epoxysuccinate WL898 (an optimised, cell-permeable E64 analogue reported as R14Et)^25^. Nevertheless, this WL898 inhibition had no noticeable effect on IPD toxicity (Figure S4). To explain this unaltered toxicity, we looked at the dynamics of cathepsin activity following preinhibition by labelling with BMV109 after adding cCTS inhibitor (Figure S5). This revealed that soon after initial cCTS inhibition, replenishment of cCTS activity is apparent and complete recovery is observed after 24 h. In an attempt to counter this replenished cCTS activity we added WL898 every 8 h to further reduce cCTS activity. Nevertheless, this still did not alter the toxicity profile of the IPDs (Figure S6). When we labelled the cathepsin activity under these optimised conditions, we again see a replenishment of cathepsin activity within 24 h, albeit reduced to about 10% compared to continuous labelling with BMV109 (Figure S7). Whether this recurring cathepsin activity, potentially in combination with a lysosomotropic effect of the primary amine-containing IPD, is responsible for the largely unaltered toxicity profile is difficult to conclude. Interestingly, target preinhibition toxicity experiments are rarely reported in prodrug literature. Instead, other control experiments are performed (Table S2). This might suggest that sustained on-target inhibition is difficult to attain. Alternatively, the cytotoxic agent could be released by off-target activity (not detectable with BMV109) or the IPD is unstable in the cell culture conditions. To exclude the latter, we assayed IPD stability in serum-containing medium at 37 °C, which displayed >95% stability for all three IPDs up to 72 h (Figure S8).

### Cytotoxic IPDs allow selective elimination of M2-polarised mouse macrophages

The most commonly reported validation of targeted drug release is the comparison of induced toxicity between cell types with differential cCTS activity. As outlined above, the majority of cCTS activity in the TME is localised in TAMs with an M2-like phenotype^10^^,^^11^. Thus, we compared cCTS activity in M1- and M2-polarised mouse bone marrow-derived macrophages (BMDMs). This showed that M2 BMDMSs have a two-fold cCTS activity compared to M1s (Figure 3(D,E)). Next, we exposed M1 and M2 BMDMs to the free cytotoxic drugs or IPDs and established EC50s with the MTT assay (Figure 3(F) and Figure S9). This showed that M2 macrophages are 4.6-fold more sensitive to Dox alone compared to M1s (EC50: M2 = 86 nM vs M1 = 392 nM). This different sensitivity could be because M2 BMDMs show a higher proliferative capacity compared to M1s (data not shown). Similarly, M2 BMDMs are more sensitive to MMAE (Figure S9). As observed in RAW264.7 macrophages (Figure 3(C)), the toxicity of IPD-Dox in M2s was reduced some 3-fold relative to free Dox. This reduction in toxicity was more than 10-fold for M1 BMDMs, increasing IPD-Dox selectivity for M2s to about 18-fold (EC50: M2 = 229 nM vs M1 = 4.17 µM). Moreover, the increased cCTS activity in M2s creates a selectivity window between M2 and M1 BMDMs, where IPD-Dox is able to selectively eliminate M2 macrophages at 1 µM, while free Dox kills both M2 and M1 at this concentration (Figure 3(G)). Because LPS-treated M1 macrophages are reported to have an increased activity of α-enolase and GAPDH (two possible off-targets of the PMK warhead) compared to M2 macrophages^24^^,^^26^^,^^27^, this cannot explain the observed selectivity window. Thus, taken together we conclude that the predominant mechanism of action of IPD-Dox selectivity for M2 macrophages is due to the increased cCTS activity compared to M1 macrophages.

## Conclusion

In conclusion, we have designed and characterised a novel self-controlled release inhibitory prodrug (IPD) strategy to simultaneously inhibit target protease activity (in this case cysteine cathepsins (cCTSs))and direct drug release to cells or environments with high target protease activity. The designed IPDs show effective cCTS dependent cargo release in *in vitro* models through the self-immolative warhead design, whereas a control warhead in which the leaving group is a non-immolative Dox conjugate rescues cell viability. We highlight the challenges with long term cell-based small molecule target pre-inhibition experiments in the context of prodrug controlled release, which are commonly overlooked or not published. Lastly, by leveraging the increased cathepsin activity in TAM-like M2-polarised BMDMs, we show that this strategy allows selective elimination of M2 over M1 macrophages. These results demonstrate the potential of the IPD strategy in general, and more specifically for modulation of the immunosuppressive TME.

Contrary to classical protease targeted prodrugs, which give rise to catalytic release of cytotoxic cargo, the IPD approach introduces an additional layer of release control by attenuating the levels of target protease trigger in response to drug release. This additional control could translate into increased therapeutic windows, in particular relevant for highly toxic drug cargo with severe side effects. This IPD approach is adaptable to other enzymatic triggers and types of cargo. For instance, switching the payload to small molecule immunostimulants could facilitate repolarisation of TAMs into an anti-tumour M1-like state. This could induce TAMs to attack tumour cells and would help repolarise immune responses in the TME. Substitution of the dipeptide target recognition motif could expand this drug delivery approach to other cysteine proteases. Furthermore, this self-controlled trigger mechanism could be applied in responsive material systems (e.g., polymer-based nanoparticles and liposomes) or as inhibitory linker system for antibody drug conjugates to further improve selective delivery.

## Supplementary Material

Supplemental Material
