# Supplementary material for: Inhibitory prodrug mechanism for cysteine cathepsin-targeted self-controlled drug release
Source: J Enzyme Inhib Med Chem. 2022 Sep 19;37(1):2566–73. doi: 10.1080/14756366.2022.2122961 (PMC9487864; doi:10.1080/14756366.2022.2122961)
Supplement: Supplemental Material [file IENZ_A_2122961_SM9621.pdf]

Inhibitory Prodrug Mechanism for Cysteine Cathepsin Targeted Self-controlled Cargo Release

Floris J. van Dalen, Martijn Verdoes*

Department of Tumor Immunology and the Institute for Chemical Immunology, Radboud Institute for Molecular Life Sciences, Radboud University Medical Centre, Nijmegen, Netherlands

* Martijn.Verdoes@RadboudUMC.nl

Information Classification: General

Supplementary tables

Table S1 | Half-maximal effective concentration (EC50) in RAW264.7 macrophages and bone marrow-derived primary, M1 and M2 macrophages.

**Compound**	**RAWs**	**M1 BMDM**	**M2 BMDM**	**Selectivity (M2/M1)**
**MMAE**	7.33 nM	N.C.[1,2]	1.9 nM[1]	
**IPD-MMAE**	32.2 nM	N.C.[1,2]	3.8 nM[1]	
**Doxorubicin**	43.4 nM	392 nM	86.0 nM	4.6-fold
**IPD-Dox**	125 nM	4.17 µM	229 nM	18.2-fold
**IPD-Ctrl**	21.4 µM	42.8 µM	78.3 µM	

[1]MMAE shows limited response in non-proliferating cells, in M2 about 25% of cells respond to MMAE in M1 no response could be noted (Figure S9). [2]N.C. = Not converged.

Information Classification: General

Table S2 | Control experiments found in literature.

Molecule	Target enzyme	*In vitro* release assay	Pre-release toxicity assay	Control compound	Target pre-inhibition release assay	Target pre-inhibition toxicity assay	Stability assay	Ref.
p-di-2-chloroethylaminophenyl-β-D-glucopyranosid) uronic acid	β-glucuronidase	+	+	-	-	-	-	1
Doxorubicin-spacer-glucuronide, Epirubicin-glucuronide	β-glucuronidase	+	-	-	-	-	Plasma, Serum, Medium	2
Paclitaxel-Glucuronide, Paclitaxel-spacer-Glucuronide	β-glucuronidase	+	+	-	-	-	-	3
dipeptidic prodrugs	Cathepsin B	+	-	-	-	-	Plasma	4–6
9-aminocamptothecin-spacer-glururonide	β-glucuronidase	+	+	-	-	-	-	7
Doxorubicin-peptide conjugates	Prostate specific antigen	+	High-vs-low expressing cells	-	-	-	Blood, Plasma	8
Doxorubicin-PABC-O-b-glucuronylcarbamate	β-glucuronidase	+	+	-	-	-	-	9
Thapsigargin-analogue peptide conjugates	Prostate specific antigen	-	+	-	-	-	-	10
Vinblastine-peptide conjugates	Prostate specific antigen	+	High-vs-low expressing cells	-	-	-	-	11
Campothecin, doxorubicin, etoposide-trimeric conjugates	Aldolase	+	+	-	-	-	-	12
Rhamnose-doxorubicin, Rhamnose- 5-fluorouracil	Rhamnosidase	+	+	-	-	-	Blood	13
linker-Phe-Lys- Doxorubicin nanoparticles	Cathepsin B	+	-	-	+	-	-	14
Doxorubicin-glucuronide albumin-conjugates	β-glucuronidase	+	+	-	-	-	-	15
Monomethyl auristatin E-glucuronide	β-glucuronidase	+	+	-	-	-	Serum	16
Ac-Phe-Lys-PABC-Dox	Cathepsin B	-	-	-	-	-	-	17
Dendrimer-GFLG-Dox	Cathepsin B	+	-	-	-	-	-	18
Doxorubicin-galactoside	Galactosidase	-	-	-	-	-	-	19
Z-Val-Cit-SN38-PEG2000	Cathepsin B	+	-	-	-	-	Water, medium	20
Monomethyl auristatin E-glucuronide albumin-conjugates	β-glucuronidase	+	+	-	-	-	-	21

Information Classification: General

Peptidomimetic antibody–drug conjugates	Cathepsin B	+	-	-	+	-	-	22
Monomethyl auristatin E-Peptide albumin conjugate	Caspase-3	+	+	-	-	-	-	23
Sunitinib-peptide conjugates	Cathepsin B	+	High-vs-low expressing cells	-	-	-	-	24
AVPIAQ- FRRG-Dox self-assembled nanoparticles	Cathepsin B	+	High-vs-low expressing cells	-	-	-	-	25
RAFT-GAGRRAAG-Doxorubicin	Cathepsin B	+	-	-	-	-	-	26
Ac-FRRG-Doxorubicin self-assembled nanoparticles	Cathepsin B	+	High-vs-low expressing cells	-	-	-	Serum	27
Albumin-linker-FRRG-Doxorubicin	Cathepsin B	+	High-vs-low expressing cells	-	-	-	MES buffer	28

(1) Wang, S.-M.; Chern, J.-W.; Yeh, M.-Y.; Ng, J. C.; Tung, E.; Roffler, S. R. Specific Activation of Glucuronide Prodrugs by Antibody-Targeted Enzyme Conjugates for Cancer Therapy. *Cancer Res.* **1992**, *52* (16), 4484–4491.

(2) Haisma, H. J.; Muijen, M. van; Pinedo, H. M.; Boven, E. Comparison of Two Anthracycline-Based Prodrugs for Activation by a Monoclonal Antibody-β-Glucuronidase Conjugate in the Specific Treatment of Cancer. *Cell Biophys.* **1994**, *24–25*, 185–192. https://doi.org/10.1007/BF02789229.

(3) de Bont, D. B. A.; Leenders, R. G. G.; Haisma, H. J.; van der Meulen-Muileman, I.; Scheeren, H. W. Synthesis and Biological Activity of β-Glucuronyl Carbamate-Based Prodrugs of Paclitaxel as Potential Candidates for ADEPT. *Bioorg. Med. Chem.* **1997**, *5* (2), 405–414. https://doi.org/10.1016/S0968-0896(96)00249-0.

(4) Dubowchik, G. M.; Firestone, R. A. Cathepsin B-Sensitive Dipeptide Prodrugs. 1. A Model Study of Structural Requirements for Efficient Release of Doxorubicin. *Bioorg. Med. Chem. Lett.* **1998**, *8* (23), 3341–3346.

(5) Dubowchik, G. M.; Mosure, K.; Knipe, J. O.; Firestone, R. A. Cathepsin B-Sensitive Dipeptide Prodrugs. 2. Models of Anticancer Drugs Paclitaxel (Taxol®), Mitomycin C and Doxorubicin. *Bioorg. Med. Chem. Lett.* **1998**, *8* (23), 3347–3352. https://doi.org/10.1016/S0960-894X(98)00610-6.

(6) Dubowchik, G. M.; Firestone, R. A.; Padilla, L.; Willner, D.; Hofstead, S. J.; Mosure, K.; Knipe, J. O.; Lasch, S. J.; Trail, P. A. Cathepsin B-Labile Dipeptide Linkers for Lysosomal Release of Doxorubicin from Internalizing Immunoconjugates: Model Studies of Enzymatic Drug Release and Antigen-Specific In Vitro Anticancer Activity. *Bioconjug. Chem.* **2002**, *13* (4), 855–869. https://doi.org/10.1021/bc025536j.

(7) Leu, Y. L.; Roffler, S. R.; Chern, J. W. Design and Synthesis of Water-Soluble Glucuronide Derivatives of Camptothecin for Cancer Prodrug Monotherapy and Antibody-Directed Enzyme Prodrug Therapy (ADEPT). *J. Med. Chem.* **1999**, *42* (18), 3623–3628. https://doi.org/10.1021/jm990124q.

(8) Garsky, V. M.; Lumma, P. K.; Feng, D. M.; Wai, J.; Ramjit, H. G.; Sardana, M. K.; Oliff, A.; Jones, R. E.; DeFeo-Jones, D.; Freidinger, R. M. The Synthesis of a Prodrug of Doxorubicin Designed to Provide Reduced Systemic Toxicity and Greater Target Efficacy. *J. Med. Chem.* **2001**, *44* (24), 4216–4224. https://doi.org/10.1021/jm0101996.

(9) Houba, P. H. J.; Boven, E.; Meulen-Muileman, I. H. van der; Leenders, R. G. G.; Scheeren, J. W.; Pinedo, H. M.; Haisma, H. J. Pronounced Antitumor Efficacy of Doxorubicin When given as the Prodrug DOX-GA3 in Combination with a Monoclonal Antibody β-Glucuronidase Conjugate. *Int. J. Cancer* **2001**, *91* (4), 550–554. https://doi.org/https://doi.org/10.1002/1097-0215(200002)9999:9999<::AID-IJC1075>3.0.CO;2-L.

(10) Jakobsen, C. M.; Denmeade, S. R.; Isaacs, J. T.; Gady, A.; Olsen, C. E.; Christensen, S. B. Design, Synthesis, and Pharmacological Evaluation of Thapsigargin Analogues for Targeting Apoptosis to Prostatic Cancer Cells. *J. Med. Chem.* **2001**, *44* (26), 4696–4703. https://doi.org/10.1021/jm010985a.

(11) Brady, S. F.; Pawluczyk, J. M.; Lumma, P. K.; Feng, D.-M.; Wai, J. M.; Jones, R.; DeFeo-Jones, D.; Wong, B. K.; Miller-Stein, C.; Lin, J. H.; Oliff, A.; Freidinger, R. M.; Garsky, V. M. Design and Synthesis of a Pro-Drug of Vinblastine Targeted at Treatment of Prostate Cancer with Enhanced Efficacy and Reduced Systemic Toxicity. *J. Med. Chem.* **2002**, *45* (21), 4706–4715. https://doi.org/10.1021/jm020139f.

(12) Haba, K.; Popkov, M.; Shamis, M.; Lerner, R. A.; Barbas, C. F.; Shabat, D. Single-Triggered Trimeric Prodrugs. *Angew. Chem. Int. Ed.* **2005**, *44* (5), 716–720. https://doi.org/10.1002/anie.200461657.

(13) Garnier, P.; Wang, X.-T.; Robinson, M. A.; Kasteren, S. van; Perkins, A. C.; Frier, M.; Fairbanks, A. J.; Davis, B. G. Lectin-Directed Enzyme Activated Prodrug Therapy (LEAPT): Synthesis and Evaluation of Rhamnose-Capped Prodrugs. *J. Drug Target.* **2010**, *18* (10), 794–802. https://doi.org/10.3109/1061186X.2010.529909.

Information Classification: General

(14) Yang, Y.; Aw, J.; Chen, K.; Liu, F.; Padmanabhan, P.; Hou, Y.; Cheng, Z.; Xing, B. Enzyme-Responsive Multifunctional Magnetic Nanoparticles for Tumor Intracellular Drug Delivery and Imaging. *Chem. – Asian J.* **2011**, *6* (6), 1381–1389. https://doi.org/https://doi.org/10.1002/asia.201000905.

(15) Legigan, T.; Clarhaut, J.; Renoux, B.; Tranoy-Opalinski, I.; Monvoisin, A.; Berjeaud, J.-M.; Guilhot, F.; Papot, S. Synthesis and Antitumor Efficacy of a β-Glucuronidase-Responsive Albumin-Binding Prodrug of Doxorubicin. *J. Med. Chem.* **2012**, *55* (9), 4516–4520. https://doi.org/10.1021/jm300348r.

(16) Legigan, T.; Clarhaut, J.; Renoux, B.; Tranoy-Opalinski, I.; Monvoisin, A.; Jayle, C.; Alsarraf, J.; Thomas, M.; Papot, S. Synthesis and Biological Evaluations of a Monomethylauristatin E Glucuronide Prodrug for Selective Cancer Chemotherapy. *Eur. J. Med. Chem.* **2013**, *67*, 75–80. https://doi.org/10.1016/j.ejmech.2013.06.037.

(17) Tang, L.; Duan, R.; Zhong, Y.; Firestone, R. A.; Hong, Y.; Li, J.; Xin, Y.; Wu, H.; Li, Y. Synthesis, Identification and in Vivo Studies of Tumor-Targeting Agent Peptide Doxorubicin (PDOX) to Treat Peritoneal Carcinomatosis of Gastric Cancer with Similar Efficacy but Reduced Toxicity. *Mol. Cancer* **2014**, *13*, 44. https://doi.org/10.1186/1476-4598-13-44.

(18) Li, N.; Li, N.; Yi, Q.; Luo, K.; Guo, C.; Pan, D.; Gu, Z. Amphiphilic Peptide Dendritic Copolymer-Doxorubicin Nanoscale Conjugate Self-Assembled to Enzyme-Responsive Anti-Cancer Agent. *Biomaterials* **2014**, *35* (35), 9529–9545. https://doi.org/10.1016/j.biomaterials.2014.07.059.

(19) Ma, Y.; Chen, H.; Su, S.; Wang, T.; Zhang, C.; Fida, G.; Cui, S.; Zhao, J.; Gu, Y. Galactose as Broad Ligand for Multiple Tumor Imaging and Therapy. *J. Cancer* **2015**, *6* (7), 658–670. https://doi.org/10.7150/jca.11647.

(20) Zhang, X.; Tang, K.; Wang, H.; Liu, Y.; Bao, B.; Fang, Y.; Zhang, X.; Lu, W. Design, Synthesis, and Biological Evaluation of New Cathepsin B-Sensitive Camptothecin Nanoparticles Equipped with a Novel Multifuctional Linker. *Bioconjug. Chem.* **2016**, *27* (5), 1267–1275. https://doi.org/10.1021/acs.bioconjchem.6b00099.

(21) Renoux, B.; Raes, F.; Legigan, T.; Péraudeau, E.; Eddhif, B.; Poinot, P.; Tranoy-Opalinski, I.; Alsarraf, J.; Koniev, O.; Kolodych, S.; Lerondel, S.; Pape, A. L.; Clarhaut, J.; Papot, S. Targeting the Tumour Microenvironment with an Enzyme-Responsive Drug Delivery System for the Efficient Therapy of Breast and Pancreatic Cancers. *Chem. Sci.* **2017**, *8* (5), 3427–3433. https://doi.org/10.1039/C7SC00472A.

(22) Wei, B.; Gunzner-Toste, J.; Yao, H.; Wang, T.; Wang, J.; Xu, Z.; Chen, J.; Wai, J.; Nonomiya, J.; Tsai, S. P.; Chuh, J.; Kozak, K. R.; Liu, Y.; Yu, S.-F.; Lau, J.; Li, G.; Phillips, G. D.; Leipold, D.; Kamath, A.; Su, D.; Xu, K.; Eigenbrot, C.; Steinbacher, S.; Ohri, R.; Raab, H.; Staben, L. R.; Zhao, G.; Flygare, J. A.; Pillow, T. H.; Verma, V.; Masterson, L. A.; Howard, P. W.; Safina, B. Discovery of Peptidomimetic Antibody–Drug Conjugate Linkers with Enhanced Protease Specificity. *J. Med. Chem.* **2018**, *61* (3), 989–1000. https://doi.org/10.1021/acs.jmedchem.7b01430.

(23) Chung, S. W.; Cho, Y. S.; Choi, J. U.; Kim, H. R.; Won, T. H.; Kim, S. Y.; Byun, Y. Highly Potent Monomethyl Auristatin E Prodrug Activated by Caspase-3 for the Chemoradiotherapy of Triple-Negative Breast Cancer. *Biomaterials* **2019**, *192*, 109–117. https://doi.org/10.1016/j.biomaterials.2018.11.001.

(24) Karnthaler-Benbakka, C.; Koblmüller, B.; Mathuber, M.; Holste, K.; Berger, W.; Heffeter, P.; Kowol, C. R.; Keppler, B. K. Synthesis, Characterization and in Vitro Studies of a Cathepsin B-Cleavable Prodrug of the VEGFR Inhibitor Sunitinib. *Chem. Biodivers.* **2019**, *16* (1). https://doi.org/10.1002/cbdv.201800520.

(25) Shim, M. K.; Moon, Y.; Yang, S.; Kim, J.; Cho, H.; Lim, S.; Yoon, H. Y.; Seong, J.-K.; Kim, K. Cancer-Specific Drug-Drug Nanoparticles of pro-Apoptotic and Cathepsin B-Cleavable Peptide-Conjugated Doxorubicin for Drug-Resistant Cancer Therapy. *Biomaterials* **2020**, *261*, 120347. https://doi.org/10.1016/j.biomaterials.2020.120347.

(26) Herceg, V.; Bouilloux, J.; Janikowska, K.; Allémann, E.; Lange, N. Cathepsin B-Cleavable Cyclopeptidic Chemotherapeutic Prodrugs. *Molecules* **2020**, *25* (18), 4285. https://doi.org/10.3390/molecules25184285.

(27) Yang, S.; Shim, M. K.; Kim, W. J.; Choi, J.; Nam, G.-H.; Kim, J.; Kim, J.; Moon, Y.; Kim, H. Y.; Park, J.; Park, Y.; Kim, I.-S.; Ryu, J. H.; Kim, K. Cancer-Activated Doxorubicin Prodrug Nanoparticles Induce Preferential Immune Response with Minimal Doxorubicin-Related Toxicity. *Biomaterials* **2021**, *272*, 120791. https://doi.org/10.1016/j.biomaterials.2021.120791.

(28) Cho, H.; Shim, M. K.; Yang, S.; Song, S.; Moon, Y.; Kim, J.; Byun, Y.; Ahn, C.-H.; Kim, K. Cathepsin B-Overexpressed Tumor Cell Activatable Albumin-Binding Doxorubicin Prodrug for Cancer-Targeted Therapy. *Pharmaceutics* **2022**, *14* (1), 83. https://doi.org/10.3390/pharmaceutics14010083.

Information Classification: General

Supplementary figures

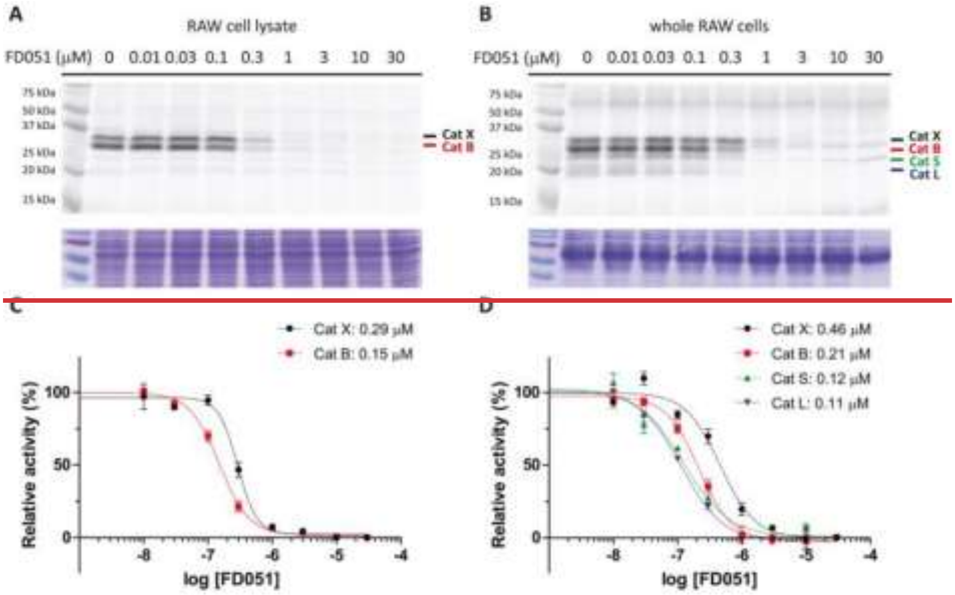

Figure S1 | cABPP and half-maximal inhibitory concentrations for compound 1 (IPD-AMC). RAW264.7 lysate (corresponding to 2·10^5 cells) (**A**) or live RAW264.7 cells (2·10^5 cells) (**B**) were incubated with indicated concentration of IPD-AMC (1 h at 37 °C) after which residual cathepsin activity was labeled with BMV109 (1 μM, 1 h at 37 °C). Cells were lysed, the lysate was cleared, proteomes were separated by SDS PAGE, and cathepsin labeling was visualised by in-gel fluorescence scanning (N=2, n=2).

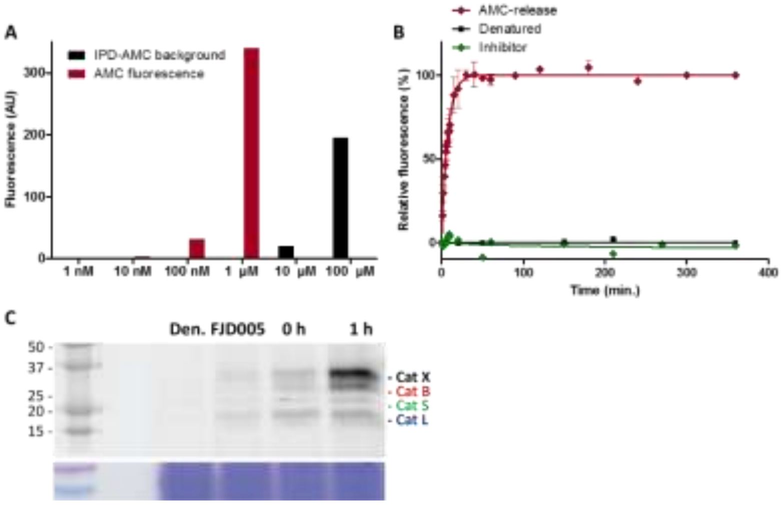

Figure S1 | Quenching efficiency and AMC release from IPD-AMC. A) Fluorescent signal for free AMC (red bars) and background fluorescence for IPD-AMC (black bars) indicating >99% quenching efficiency of the AMC fluorescence in intact IPD-AMC. **B)** AMC release over time in RAW264.7 lysate and deactivated RAW264.7 lysate (denatured or inhibited) indicates AMC is released in a cathepsin-dependent manner and shows no release over extended time in deactivated lysate (6 h at 37 °C) (n = 3). **C**) In tandem (c)ABPP for experiment Fig. 2B: RAW264.7 lysate was either pretreated by denaturation (Den., 95 °C, 5 min) or inhibition with inhibitor FJD005 (10 µM, 37 °C for 5 min), followed by labeling with BMV109. BMV109 in RAW264.7 lysate (at t = 0) serves as background control.

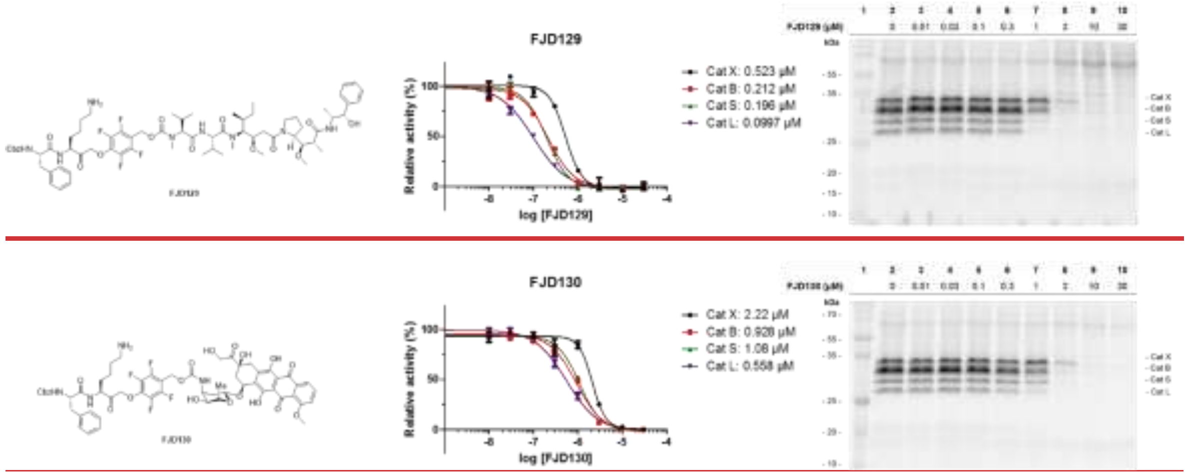

Figure S3 | cABPP and half-maximal inhibitory concentrations for compound 2 (FJD129) and 3 (FJD130). RAW264.7 lysate (corresponding to 2·10⁵ cells) or live RAW264.7 cells (2·10⁵ cells) were incubated with indicated concentration of **2** or **3** (1 h at 37 °C) after which residual cathepsin activity was labeled with BMV109 (1 µM, 1 h at 37 °C). Cells were lysed, the lysate was cleared, proteomes were separated by SDS PAGE, and cathepsin labeling was visualised by in-gel fluorescence scanning (N=2, n=2).

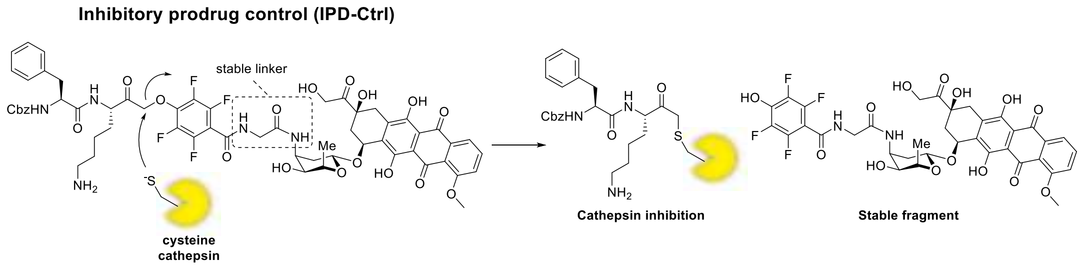

Figure S2 | Design of an IPD control. By introduction of a stable glycine linker the electronic cascade is interrupted and release of active doxorubicin is prevented.

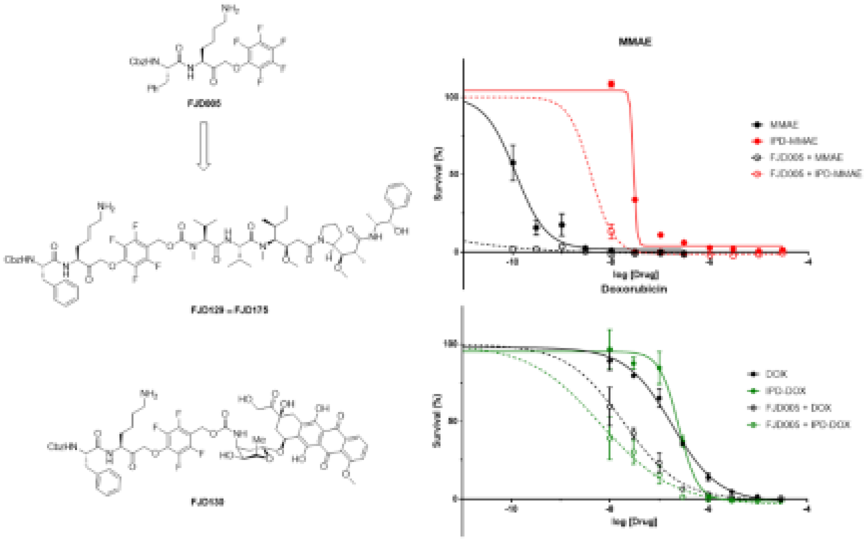

Figure S3 | Preinhibition MTT assay with broad-spectrum inhibitor FJD005. RAW264.7 macrophages were treated with FJD005 (1 µM) or vehicle for 1 h after which the cells were treated with the indicated concentration of drug for 3 days. Cell viability was assessed with an MTT assay (N=1, n=2). Preinhibition was unable to rescue cell proliferation, instead, preinhibition displayed enhanced cell killing for both IPD and free drugs.

Information Classification: General

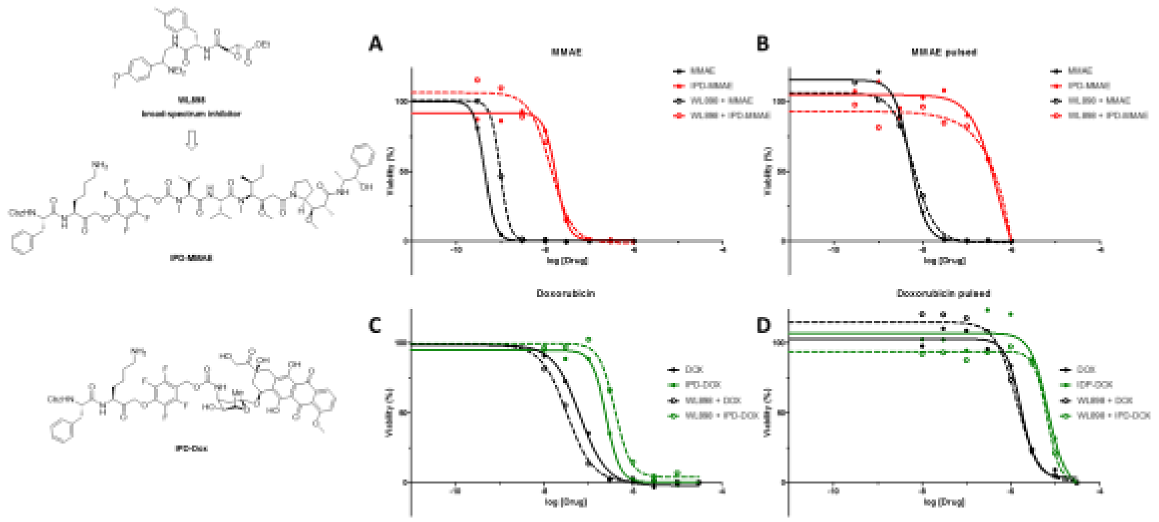

Figure S4 | Preinhibition MTT assay with broad-spectrum inhibitor WL898. A, C) RAW264.7 macrophages were treated with WL898 (1 µM) or vehicle for 1 h after which the cells were treated with the indicated concentration of drug for 3 days. Cell viability was assessed with an MTT assay. Preinhibition was unable to rescue cell proliferation. We hypothesized that this might be due to recovery of cathepsin activity over time. To avoid recurring cathepsin activity we next attempted to limit drug release to the first hour of cathepsin inhibition, as this would then allow drug release for the vehicle-treated sample while the in the pre-inhibited conditions the cathepsins would be inactive. Thus, we pulsed cells for 1 hour with WL898 or vehicle followed by 1 hour pulsing with IPD or cytotoxic agent.

B, D) RAW264.7 macrophages were treated with WL898 (1 µM) or vehicle for 1 h after which the cells were treated with the indicated concentration of drug for 1 h and the medium was refreshed (2x). Cells were incubated for 3 days and cell viability was assessed with an MTT assay. Pulsing cells with inhibitor and IPD equally reduced cytotoxicity in all conditions. Most likely this is due to cells taking up similar amounts of unprocessed IPD, prior to washing away the excess. Recurring cathepsin activity is then able to release equal amounts of drug in vehicle-treated and pre-inhibited conditions alike.

Information Classification: General

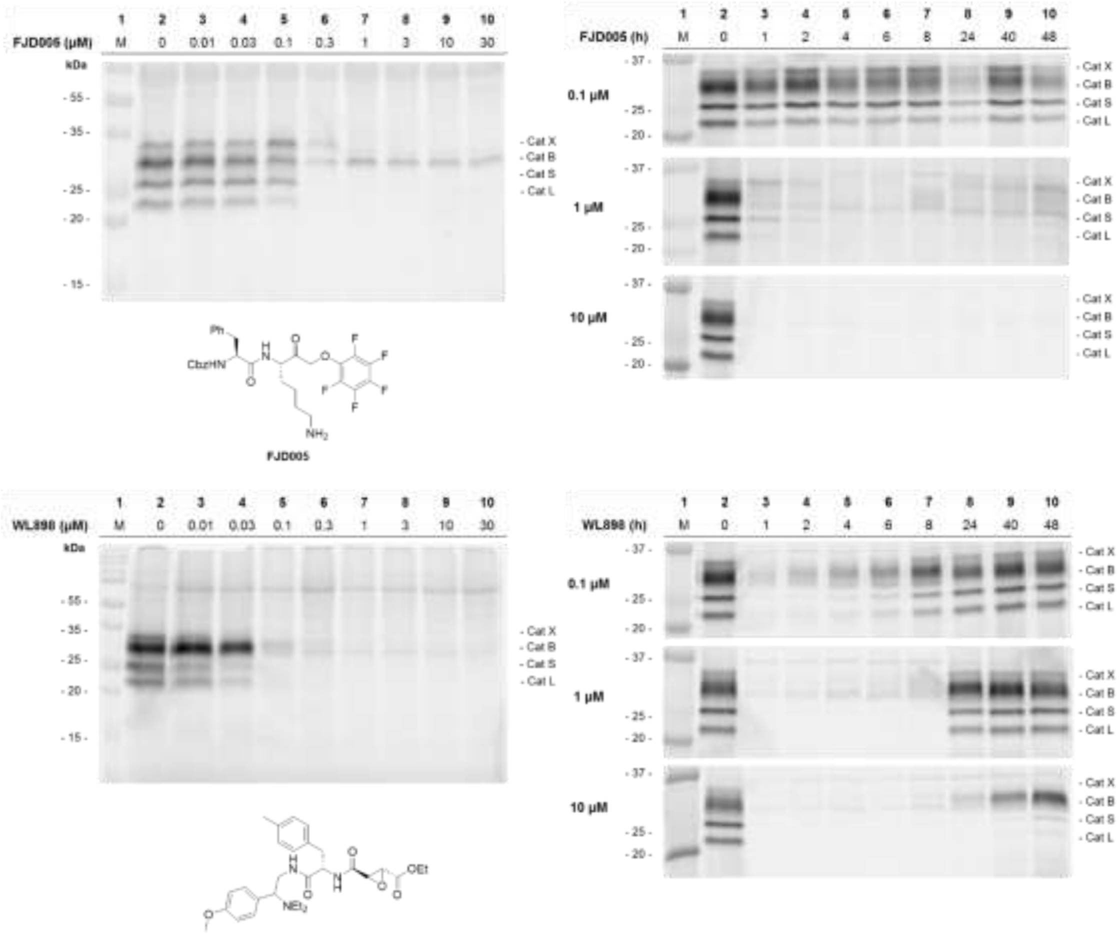

Figure S5 | cABPP of FJD005 and WL898 over time in RAW264.7 macrophages. Live RAW264.7 cells ($2{\cdot}10^5$ cells) were incubated with indicated concentration of inhibitor (1 h or indicated time point at 37 °C) after which residual cathepsin activity was labeled with BMV109 (1 µM, 1 h, 37 °C). Cells were lysed, the lysate was cleared, proteomes were separated by SDS PAGE, and cathepsin labeling was visualised by in-gel fluorescence scanning (N=1, n=2). Cathepsin activity replenishes over time either due to cell proliferation or resynthesis of cathepsins. FJD005 displayed toxicity at 10 µM.

Information Classification: General

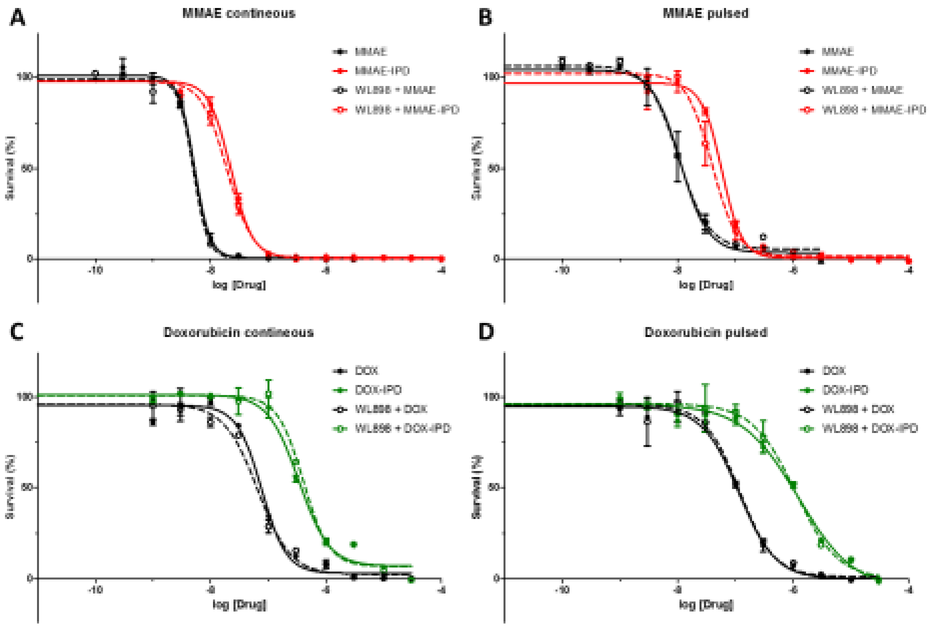

Figure S6 | Preinhibition MTT assay with broad-spectrum inhibitor WL898 every 8 hours. A, C) RAW264.7 macrophages were treated with WL898 (1 µM) or vehicle for 1 h after which the cells were treated with the indicated concentration of drug for 3 days, during which WL898 (1 µM) was added every 8 h to keep cathepsins inhibited. Cell viability was assessed with an MTT assay (N=1, n=3). **B, D)** RAW264.7 macrophages were treated with WL898 (1 µM) or vehicle for 1 h after which the cells were treated with the indicated concentration of drug for 7 h and this was repeated every 8 h. Cells were incubated for a total of 3 days and cell viability was assessed with an MTT assay (N=1, n=3). Preinhibition supplemented with WL898 every 8 hours was unable to rescue cell proliferation. Pulsing cells every 8 hours equally reduced toxicity in all conditions measured similar to the previous experiment.

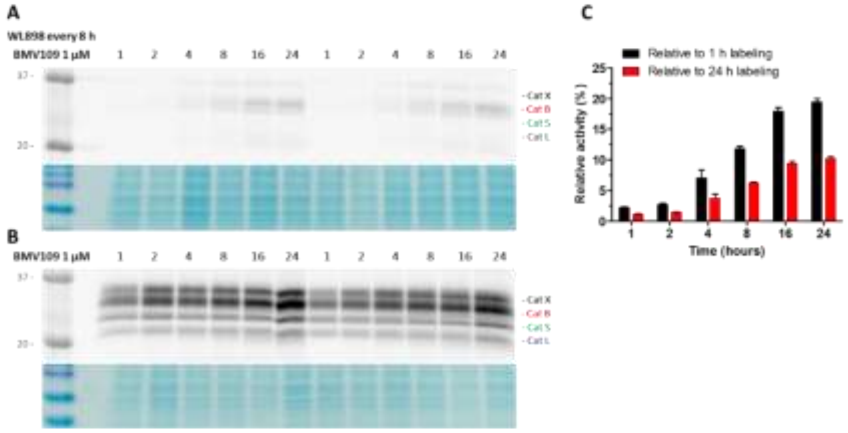

Figure S7 | cABPP with after inhibition by WL898 every 8 hours in RAW264.7 macrophages. A) Live RAW264.7 cells ($2 \cdot 10^5$ cells) were incubated with 1 µM WL898 (200x in DMSO) for 1 h at 37 °C after which 1 µM BMV109 (200x in DMSO) was added and the cells were incubated for the indicated time point at 37 °C (WL898 was added every 8 hours to keep cathepsins inhibited). Cells were lysed, the lysate was cleared, proteomes were separated by SDS PAGE, and cathepsin labeling was visualised by in-gel fluorescence scanning (N=1, n=2). **B)** Live RAW264.7 cells ($2 \cdot 10^5$ cells) were incubated with 1 µM BMV109 (200x in DMSO) for the indicated time point. Cells were lysed, the lysate was cleared, proteomes were separated by SDS PAGE, and cathepsin labeling was visualised by in-gel fluorescence scanning (N=1, n=2). **C)** Cathepsin activity was quantified and scaled to 1 h labeling (black) or 24 h continuous labeling (red). Cathepsin activity replenishes over time even though WL898 is refreshed every 8 hours. This results in recurring cathepsin activity which can be quantified as ~20% relative to 1 hour labeling or ~10% relative to 24 hour uninterrupted labeling.

Information Classification: General

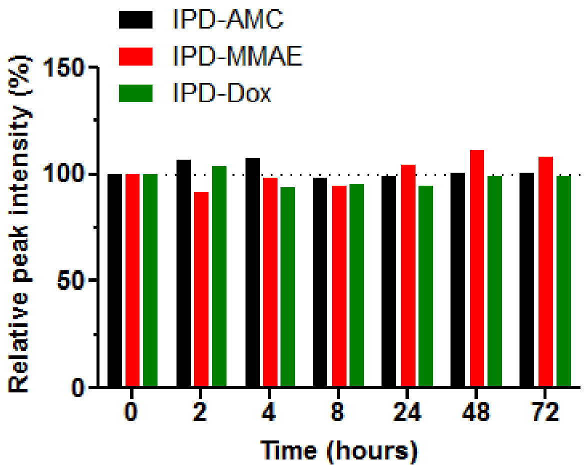

Figure S8 | Stability of IPD-AMC, IPD-MMAE and IPD-Dox in conditioned medium. IPDs (100 µM from 200x DMSO stock) were taken up in complete medium (DMEM supplemented with 10% foetal calf serum and 1/100 anti-anti) and incubated at 37 °C for 72 hours. At the indicated time point aliquots (20 µl) were drawn and diluted with cold, acidified acetonitrile (40 µL, 0.1% TFA in MeCN). The samples were centrifuged (21 130x g for 15 min at 4 °C) and the supernatant was injected into the HPLC. A peak of the conditioned medium was selected as internal standard. Data was integrated, corrected for internal standard and scaled to 0 h as 100% reference point (n=1).

Information Classification: General

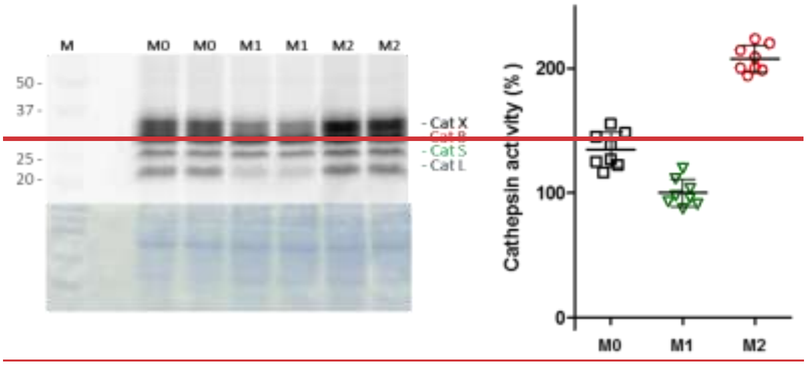

~~**Figure S9 | Cathepsin activity in polarized BMDMs.** M0, M1 or M2 BMDMs (some 2·10^5) were treated with 1 µM BMV109 (200x in DMSO) for 1 hour at 37 °C. Cells were lysed, the lysate was cleared, proteomes were separated by SDS PAGE, and cathepsin labeling was visualised by in-gel fluorescence scanning (n=8). The labeling was quantified with ImageJ software, corrected for background and scaled to M1 as 100% reference point. M2 BMDMs display some 2-fold increase in cathepsin activity compared to M1 macrophages (n=8).~~

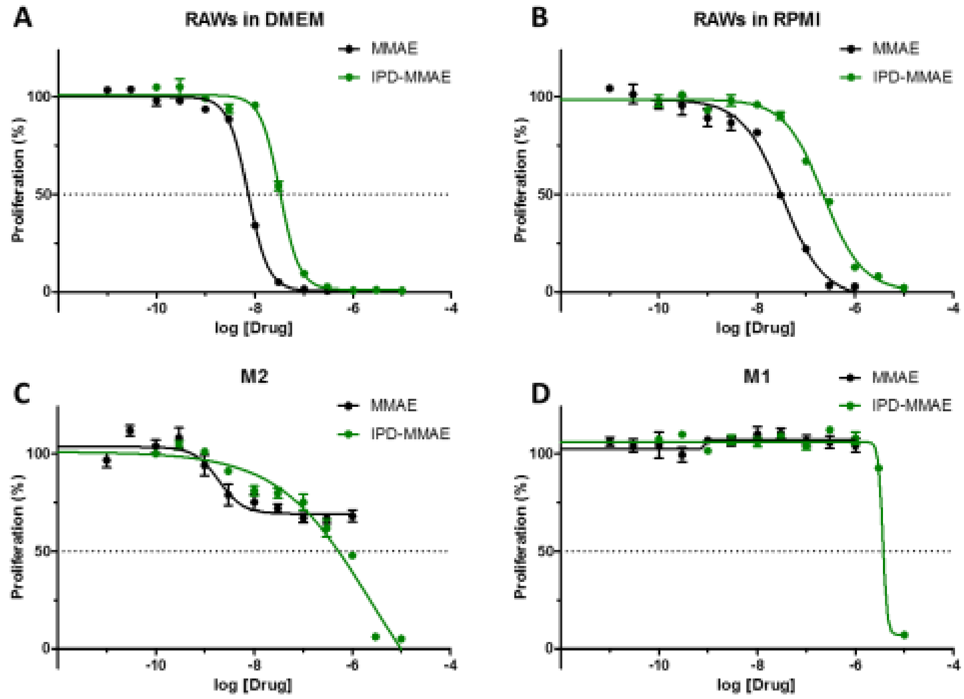

Figure S9 | MTT assay for MMAE and IPD-MMAE on RAW264.7 macrophages and BMDMs. A, B) RAW264.7 macrophages were treated with indicated concentration of MMAE or IPD-MMAE for 3 days, after which cell viability was assessed with an MTT assay. **C, D)** Bone marrow-derived macrophages were polarized into M1 or M2 populations and were treated with indicated concentrations of MMAE or IPD-MMAE for 3 days, after which cell viability was assessed by MTT assay. RAWs cultured in DMEM (optimal medium) are more sensitive to MMAE than RAWs in RPMI (suboptimal medium). MMAE shows partial toxicity in M2 and no activity M1 BMDMs.

Supplementary schemes

Scheme S1 | Synthesis of control compound **23** (IPD-Ctrl).

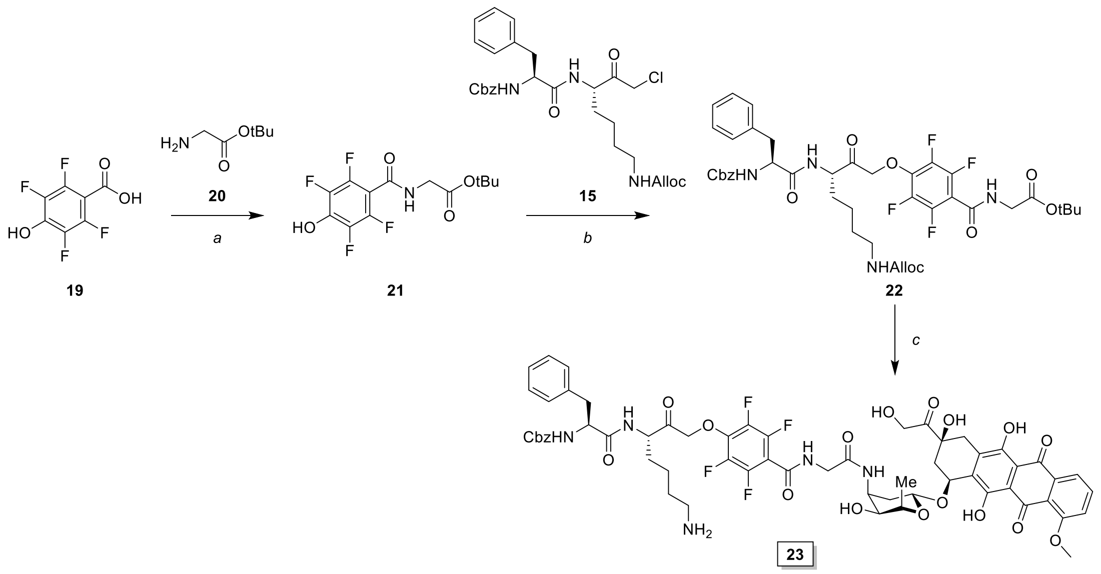

Reagents and conditions: **a) 20**, EDCI, HOBt, NMM, DMF, r.t., 16 h, 90%, **b) 15**, KF, DMF, 60 °C, 16 h, 90%, **c)** *i.* TFA/DCM (1:1, v:v), r.t., 2.5 h, *ii.* Doxorubicin·HCl, EDCI, HOBt, DiPEA, DMF, 16 h, r.t., *iii.* Pd(PPh$_3$)$_4$, DMBA, DCM, r.t., 5 min, 52% over 3 steps.

Information Classification: General

Experimental procedures

General remarks | All commercial chemical reagents were used as received unless indicated otherwise. Water-sensitive reactions were performed in anhydrous solvents in flame-dried glassware under an overflow of argon gas. Dichloromethane (DCM), diethyl ether (Et_2O), tetrahydrofuran (THF), acetonitrile (MeCN) and toluene were dried by purging over activated alumina columns in an MBraun MB SPS800 and stored over activated 3 Å molecular sieves. Technical grade n-heptane was distilled prior to use. Trimethylamine (Et_3N) and N,N-diisopropylethylamine (DiPEA) were distilled over calcium hydride and stored over potassium hydroxide pellets. Reactions were monitored using analytic thin-layer chromatography (TLC) on glass TLC plates (60G, F_{254}, Merck) with UV detection at 254 nm and by staining with either a solution of Baeyer's reagent ($KMnO_4$ (10 g/l) and K_2CO_3 (50 g/l) in water) or by staining with a ninhydrin-solution (10 g/l) in BuOH/AcOH (99/1; v/v) and subsequent charring at ~150 °C. Flash column chromatography was carried out on SiliaFlash® silica gel (40 – 63 µm, 60 Å, SiliCycle). Analytic LC/MS was performed using a hybrid Thermo Finnigan/Shimadzu system equipped with a Phenomenex Gemini NX C18 column (3 µm, 110 Å, 50 mmL x 2.0 mmD) using mobile phases A: 1% aq. HCOOH, B: 1% HCOOH in MeCN, with detection at 210 – 600 nm by a diode array, coupled to a LCQ fleet mass spectrometer (Thermo Finnigan). Preparative HPLC was performed using a Shimadzu system equipped with a Phenomenex Gemini NX-C18 column (5 µm, 110 Å, 150 mmL x 21.2 mmD) in combination with mobile phases A: 0.1% TFA in H_2O, B: 0.1% TFA in MeCN, with detection at 214/254 nm. Electrospray ionisation (ESI) mass spectrometry (MS) was performed on a LCQ Advantage Max (Thermo Finnigan). High resolution mass spectrometry (HRMS) (ESI-TOF) was recorded on a AccuTOF CS JMS-T100CS mass spectrometer (JEOL), equipped with an electrospray ion source in positive mode (source voltage 2.4 kV, capillary temperature 250 °C) with resolution R = 7000 (mass range = 10 – 10 000). ^{1}H NMR and ^{13}C NMR spectra were recorded on a Brüker Avance III 400 (9.4 T) or Brüker Avance III 500 (11.7 T) spectrometer. Chemical shifts (δ) are reported in ppm relative to tetramethylsilane as internal standard or the residual signal of the deuterated solvent was used as reference point. Coupling constants (J) are given in Hz. All ^{13}C APT experiments were proton decoupled.

Cell culture | RAW264.7 cells (264.7) were maintained in DMEM (Gibco®) containing high glucose, stable glutamine (GlutaMAX™), sodium pyruvate and phenol red, which was supplemented with 10% foetal calf serum (FCS, Bio-Greiner One) and antibiotics (100 units/ml penicillin, 100 µg/ml streptomycin, and 250 ng/ml amphotericin B (Gibco®)). The cells were cultured in T75 flask (Corning) in a humidified 5% CO_2-atmosphere at 37 °C and the culture was passaged every 2-3 days. The cells were suspended via cell scraper, followed by centrifugation at 1000x *g* for 5 min at 4 °C. The medium was refreshed and the cells were seeded to the appropriate confluence. Cells were harvested at 80-90% confluence.

BMDM preparation | Mouse bone marrow was isolated from the femur and tibia from 8-12 weeks old female wild type C57BL/6J mice (Charles River, France). In short, hind legs were dissected and muscle tissue was removed. Bones were cleaned in 70% ethanol for 3 min, washed with PBS and conditioned medium; RPMI1640 (Gibco) containing HEPES and phenol red, which was supplemented with FCS (10%), antibiotics (100 units/ml penicillin, 100 µg/ml streptomycin, and 250 ng/ml amphotericin B (Gibco)), 2 mM ultraglutamine (Lonza) and 50 µM β-mercaptoethanol (Sigma Aldrich, freshly added from 50 mM aliquots prepared under oxygen-poor conditions and stored at -20 °C). Bones were cut with a scalpel, marrow was flushed out with 5-10 ml complete medium and filtered over 100 µm mesh strainer (Corning). The cells were centrifuged at 1000x *g* for 5 min at 4 °C and the red blood cells were lysed with ACK lysis buffer (150 mM NH_4Cl, 10 mM $KHCO_3$, 0.1 mM disodium

Information Classification: General

EDTA) 30 s on ice. 50 ml PBS was added and cells were centrifuged at 1000x *g* for 5 min at 4 °C. Cells (2·10^6) were seeded in a non-culture treated 10 cm PS petridish (Falcon) and bone marrow-derived macrophages were obtained by differentiation under influence of recombinant mouse 20 ng/ml M-CSF (all mouse cytokines were sourced from Peprotech) in 10 ml complete medium. The cells were cultured in a humidified 5% CO_2-atmosphere at 37 °C and the medium was supplemented on day 3 with 5 ml complete medium containing 20 ng/ml M-CSF. On day 5, the medium was refreshed and cells were activated to form M0 (20 ng/ml M-CSF), M1 (20 ng/ml GM-CSF, 50 ng/ml IFNγ and 100 ng/ml LPS) and M2 (20 ng/ml M-CSF and 10 ng/ml IL-4) populations. At day 6 the floating population (<5% of cells) was removed by washing with PBS and the adherent population was harvested via cell scraper.

Lysate preparation | Cellular lysate was prepared from RAW264.7 culture harvested at 70-90% confluence. The cells were suspended, per described method, followed by centrifugation at 1000x g for 5 min at 4 °C. The supernatant was removed and the pellet was suspended in 10 µl per 1·10^6 cells citrate buffer (50 mM citric acid pH 5.5, 5 mM DDT, 0.5% CHAPS, and 0.1% Triton X-100). The mixture was put on ice for 15 min, sonicated 3x 5 seconds on ice, followed by centrifugation at 21 130x g for 15 min at 4 °C. The cleared lysate was transferred into pre-cooled Eppendorf's (0.5 ml aliquots) and stored at -20 °C.

cABPP in whole cells or lysate | RAW264.7 macrophages or BMDMs (some 2·10^5 cells in 100 µl conditioned medium) were incubated with the indicated concentration of inhibitor (200x in DMSO) for 1 hour at 37 °C, followed by labeling with 1 µM BMV109 (200x in DMSO) for 1 hour at 37 °C. The cells were centrifuged at 10 000x *g* for 1 min at r.t., the supernatant was removed, and the cells were taken up in 9 µl hypotonic lysis buffer (50 mM PIPES pH 7.4, 10 mM KCl, 5 mM MgCl, 4 mM DTT, 2 mM EDTA, and 1% NP40). The lysate was incubated on ice for 5 min, followed by centrifugation at 21 130x *g* for 15 min at 4 °C. The cleared lysate was diluted with 3 µl Laemmli's 4x sample buffer (40% glycerol, Tris/HCl (0.2 M, pH 6.8), 8% SDS, 10% BME, and 0.04% bromophenol blue) and the mixture was denatured over 5 min at 95 °C. The samples were spun down and separated by SDS PAGE (15%, 15 min at 80 V, 1.5-2 hours at 120 V). The gel was analysed by in-gel fluorescence scanning on a Typhoon Trio flat-bed laser scanner (GE Healthcare) and equal protein loading was confirmed by staining with Coomassie® Brilliant Blue R-250 (Schmidt GmbH).

RAW264.7 lysate (10 µl, in citrate buffer pH 5.5) was incubated with the indicated concentration of inhibitor (20x in citrate buffer) for 1 hour at 37 °C, followed by labeling with 1 µM BMV109 (20x in citrate buffer) for 1 hour at 37 °C. The solution was centrifuged (15 min, 21 130x g, 4 °C), transferred to clean Eppendorf's and 4x sample buffer (3 µl) was added. The samples were denatured over 5 min at 95 °C and separated by SDS PAGE (15%, 15 min at 80 V, 1.5-2 hours at 120 V, 4 °C). The gel was imaged on a Typhoon Trio (GE Healthcare), and constant protein loading was confirmed by staining with Coomassie® Brilliant Blue R-250 (Schmidt GmbH).

cABPP-labeling intensities were quantified using Image J software. Data was transferred to Microsoft Excel, corrected for background fluorescence, and scaled to the positive control (DMSO, BMV109) as 100%-activity reference point. The mean, standard deviation (SD), and standard error of the mean (SEM) were calculated and normalised to the corrected positive control. The data was transferred to Graphpad Prism 6.0 and IC50-values were calculated using non-linear regression.

MTT assay | RAW264.7 cells (some 5·10^3 cells) or BMDMs (some 5·10^4 cells) were seeded in a flat-bottom 96-well plate (Bio-Greiner one). The cells were incubated with the indicated concentration of inhibitor or vehicle

Information Classification: General

for 1 hour at 37 °C after which the cells were treated with the indicated compounds for 3 days in a humidified 5% CO_2-atmosphere incubator at 37 °C. The medium was replaced with 60 µl conditioned medium and cells were incubated with 10 uL MTT (3-(4,5-Dimethylthiazol-2-yl)-2,5-Diphenyltetrazolium Bromide) (Sigma-Aldrich) solution (4 mg/ml) for 1 h at 37 °C. The medium was removed and the formed formazan crystals were dissolved in 100 µl acidic lysis buffer (90% isopronanol, 0.1% SDS, 40 mM HCl in water) for 1 hour at 37 °C. The OD595 of the dissolved crystals was measured with a BioRAD iMark microplate absorbance reader.

AMC-release assay | RAW264.7 cell lysate (20 µl, corresponding to some $2{\cdot}10^6$ cells, pH 5.5) was treated with the indicated concentration of inhibitor (40x in citrate buffer) or vehicle for 1 hour at 37 °C, followed by incubation with the indicated concentration of IPD-AMC (40x in citrate buffer) for 1 hour at 37 °C. Experiments without pretreatment were directly incubated with IPD-AMC (40x in citrate buffer) for 1 hour at 37 °C. Citrate lysis buffer incubated with IPD-AMC served as internal control. The fluorescent signal (λ_{ex} = 360±5 nm, λ_{em} = 440±5 nm, r.t.) was measured on a LS 55 spectrophotometer (Perkin Elmer).

Time-dependent labeling in RAW264.7 lysate | RAW264.7 cell lysate (from $2{\cdot}10^6$ cells) was incubated with IPD-AMC (2.5 µM) for the indicated time periods. A sample (2.5 µl) of the incubated lysate was transferred to BMV109 (2.78 µM, 22.5 µl) to quench the inhibition reaction and label residual cathepsin activity with BMV109. The resulting mixture was incubated for 1 hour at 37 °C. The proteome was cleared by centrifugation (15 min, 21 130x *g*, 4 °C), the supernatant was sampled (9 µl), and diluted with 4x sample buffer (3 µl). The proteins were denatured for 5 min at 95 °C and were resolved on SDS PAGE. The labeling intensities were imaged by in-gel fluorescence scanning on a Typhoon Trio (GE Healthcare) and labeling was quantified with Image J Software.

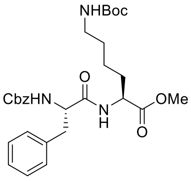

Z-FK(Boc)-OMe (6). Z-L-Phe-OH (1.5 g, 5.0 mmol) was dissolved in dry DMF (20 ml, 0.25 M) and H-L-Lys(Boc)-OMe·HCl (1.5 g, 5.0 mmol, 1 eq.), HBTU (2.1 g, 5.5 mmol, 1.1 eq.) and DiPEA (2.62 ml, 15 mmol, 3 eq.) were added. The mixture was stirred for 3 hours at r.t. and the reaction was diluted with EtOAc, washed with 1 N HCl, sat. $NaHCO_3$ and sat. NaCl, dried over Na_2SO_4, filtered, and concentrated. Flash column chromatography (30 to 60% EtOAc in heptane) afforded the title compound as white solid (2.87 g, 5.3 mmol, quant.). R_f = 0.5 (60% EtOAc in heptane); ^{1}H NMR (400 MHz, $CDCl_3$) δ 7.39 – 7.13 (m, 10H), 6.34 (d, *J* = 7.9 Hz, 1H), 5.38 (s, 1H), 5.09 (s, 2H), 4.64 (s, 1H), 4.52 (q, *J* = 7.3 Hz, 1H), 4.43 (t, *J* = 7.2 Hz, 1H), 3.70 (s, 3H), 3.15 – 3.02 (m, 4H), 1.78 (dd, *J* = 9.4, 5.7 Hz, 1H), 1.62 (s, 3H), 1.43 (s, 9H), 1.31 – 1.14 (m, 2H); ^{13}C NMR (101 MHz, $CDCl_3$) δ 172.1, 170.6, 154.9, 136.2, 135.7, 129.3, 128.7, 128.5, 128.2, 128.1, 127.1, 67.1, 56.1, 52.4, 52.0, 40.1, 31.9, 29.3, 28.4, 22.1.

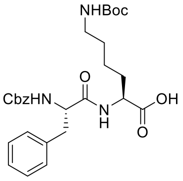

Z-FK(Boc)-OH (7). Z-FK(Boc)-OMe (2.87 g, 5.3 mmol) was dissolved in THF/MeOH/H_2O (3:1:1, v:v:v, 25 ml, 0.2 M) and to this was added lithium hydroxide monohydrate (420 mg, 10 mmol, 2 eq.) at 0 °C. The reaction was stirred for 1 hour warming to r.t. and was concentrated *in vacuo*. The reaction mixture was diluted with water, acidified with 1 N HCl to pH 3, extracted with EtOAc (3x) and the organics were collected. The extracts were washed with sat. NaCl, dried over Na_2SO_4, filtered, and concentrated *in vacuo*. Flash column chromatography (50 to 75% EtOAc/heptane) afforded the title compound as white foam (2.61 g, 5 mmol, quant.). R_f = 0.4 (60% EtOAc/heptane). ^{1}H NMR (400 MHz, $CDCl_3$) δ 7.37 – 7.09 (m, 10H), 6.76 (s, 1H), 6.24 (s, 1H), 5.03 (bs, 2H), 4.75 (s, 1H), 4.51 (s, 1H), 3.06 (s, 4H), 1.78 (d, *J* = 63.2 Hz, 2H), 1.42 (s, 11H), 1.32 – 1.18 (m, 2H).

Information Classification: General

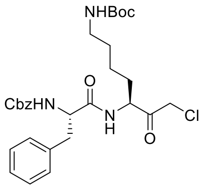

Z-FK(Boc)-CMK (8).

Of note: diazomethane is produced and utilised during this reaction, appropriate equipment and careful handling are required. Diazomethane is a friction-sensitive, explosive and toxic compound.

Z-FK(Boc)-OH (1.10 g, 2.1 mmol) was dissolved in anhydrous THF (10 ml) and cooled to -15 °C. *N*-methyl morpholine (286 µl, 2.6 mmol, 1.25 eq.) and isobutyl chloroformate (324 µl, 2.5 mmol, 1.2 eq.) were added and the reaction was stirred for 30 min at -15 °C. Meanwhile, diazomethane was prepared from N,4-dimethyl-N-nitrosobenzenesulfonamide (Diazald) (2.14 g, 10 mmol, 5 eq.). The Diazald was dissolved in Et_2O (30 ml) and cooled to 0 °C. Potassium hydroxide (0.45 g, 8 mmol, 4 eq. in 10 ml ethanol) was added, and the resulting mixture was warmed to reflux (over a 65 °C water bath). The formed ethereal solution was poured into the mixed anhydride. The reaction was stirred for 3 hours at -15 °C. Concentrated hydrogen chloride (37%, 12 M, 3 ml) in glacial acetic acid (3 ml) was added to the mixture and stirring was continued for 10 min and the reaction was poured into water. The aqueous work up was extracted with EtOAc (3x), the organics were combined, washed with sat. NaCl, dried over Na_2SO_4, filtered, and concentrated. Flash column chromatography (1 to 10% acetone in DCM), followed by recrystallisation from hot EtOAc/heptane afforded the title compound as white crystals (985 mg, 1.76 mmol, 85%). R_f = 0.5 (40% EtOAc in heptane); ^{1}H NMR (500 MHz, $CDCl_3$) δ 7.40 – 7.25 (m, 9H), 7.23 – 7.19 (m, 2H), 6.54 (d, J = 7.4 Hz, 1H), 5.43 (s, 1H), 5.11 (s, 2H), 4.77 – 4.64 (m, 2H), 4.47 (q, J = 7.3 Hz, 1H), 4.10 (s, 2H), 3.15 – 3.01 (m, 4H), 1.90 – 1.79 (m, 1H), 1.60 – 1.51 (m, 1H), 1.45 (s, 11H), 1.22 (s, 2H); ^{13}C NMR (126 MHz, $CDCl_3$) δ 200.4, 171.2, 156.2, 136.0, 129.3, 128.8, 128.6, 128.3, 128.1, 127.3, 79.3, 67.2, 56.2, 55.9, 46.4, 39.8, 38.1, 30.5, 29.4, 28.4, 22.1.

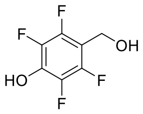

2,3,5,6-tetrafluoro-4-(hydroxymethyl)phenol (9). 2,3,5,6-tetrafluoro-4-hydroxybenzoic acid hydrate (1.098 g, 5.23 mmol) was co-evaporated with anhydrous toluene (3x) and dissolved in anhydrous THF (5 ml, 1 M). BH_3·THF solution (20 ml, 20 mmol, 1 M, 4 eq.) was added dropwise and the reaction was heated to reflux for 16 hours. The reaction mixture was quenched with 2 N HCl, diluted with water and extracted with EtOAc (3x). The organics were combined, washed with sat. NaCl, dried over Na_2SO_4, filtered, and concentrated under reduced pressure. Flash column chromatography (20 to 40% EtOAc in heptane) afforded the title compound as white solid (1.00 g, 5.10 mmol, 98%). R_f = 0.5 (40% EtOAc in heptane); ^{1}H NMR (400 MHz, Acetone-D6) δ 4.68 (t, *J* = 1.8 Hz, 2H); ^{13}C NMR (101 MHz, Acetone-D6) δ 145.4 (dddd, *J* = 243.2, 11.2, 9.8, 4.2 Hz), 137.9 (ddt, *J* = 240.6, 16.1, 4.4 Hz), 135.9 (tt, *J* = 14.3, 4.4 Hz), 109.2 (tt, *J* = 18.7, 1.4 Hz), 51.09 (tt, *J* = 3.7, 1.9 Hz).

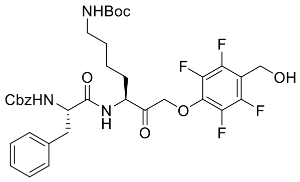

Z-FK(Boc)-PMK-OH (10). Z-FK(Boc)-CMK (166 mg, 0.30 mmol) was dissolved in anhydrous DMF (6 ml, 0.05 M), and 2,3,5,6-tetrafluoro-4-(hydroxymethyl)phenol (70 mg, 0.36 mmol, 1.2 eq.) and potassium fluoride (26 mg, 0.44 mmol, 1.5 eq.) were added. The mixture was stirred for 16 hours at 60 °C, diluted with water and the aqueous layer was extracted with EtOAc (3x). The organics were washed with sat. NaCl, dried over Na_2SO_4, filtered, and concentrated *in vacuo*. Flash column chromatography (10 to 30% EtOAc in heptane) afforded the title compound as white solid (169 mg, 0.24 mmol, 80%). R_f = 0.6 (60% EtOAc in heptane); ^{1}H NMR (500 MHz, $CDCl_3$) δ 7.42 – 7.14 (m, 11H), 6.65 (s, 1H), 5.49 (s, 1H), 5.09 (s, 2H), 4.84 (s, 2H), 4.78 (s, 2H), 4.77 – 4.73 (m, 1H), 4.48 (q, *J* = 7.4 Hz, 1H), 3.13 – 2.99 (m, 4H), 1.92 – 1.82 (m, 1H), 1.59 – 1.50 (m, 1H), 1.44 (s, 11H), 1.33 – 1.17 (m, 2H); ^{13}C NMR (126 MHz, $CDCl_3$) δ 202.7, 171.3, 156.2, 146.4, 144.4, 141.3,

139.2, 136.2, 136.1, 136.0, 129.3, 128.7, 128.5, 128.3, 128.1, 127.2, 112.8, 79.3, 75.4, 67.2, 56.2, 55.2, 52.5, 39.9, 38.1, 30.2, 29.4, 28.4, 22.2.

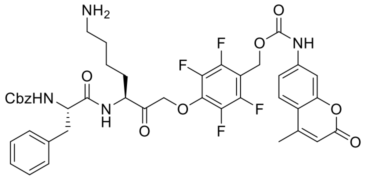

Z-FK-PMK-AMC (1, IPD-AMC). 7-amino-4-methylcoumarine (131 mg, 0.75 mmol, 5 eq.) was suspended in a 20% phosgene solution in toluene (3 ml, 1.8 M, 4.5 mmol, 30 eq.) and the reaction was stirred for 16 hours at reflux. The solution was cooled to r.t., purged with argon to remove residual phosgene the reaction was concentrated to dryness. Z-FK(Boc)-PMK-OH (107 mg, 0.15 mmol) and the intermediate isocyanate were dissolved in THF (2 ml), dibutyltin dilaurate (4.43 µl, 0.0075 mmol, 0.05 eq.) was added and the reaction was stirred for 16 hours at r.t.. The mixture was concentrated *in vacuo*, dissolved in DCM, and washed with 1 N HCl (3x), sat. $NaHCO_3$ (3x), water (3x), and sat. NaCl, dried over Na_2SO_4, filtered, and concentrated. A quarter of the crude intermediate was dissolved in TFA/DCM (1:1, v:v, 2 ml), stirred for 30 min at r.t., and the mixture was co-evaporated with toluene (3x). Preparative HPLC and consecutive lyophilisation (2x) afforded the title compound as white powder (7.8 mg, 0.0095 mmol, 25% over 2 steps). ^{1}H NMR (400 MHz, CD_3OD+D_2O) δ 7.66 – 7.54 (m, 2H), 7.36 – 7.08 (m, 10H), 6.18 (t, *J* = 1.4 Hz, 1H), 5.31 (s, 2H), 5.01 (dd, *J* = 13.9, 5.4 Hz, 2H), 4.75 (dd, *J* = 31.1, 13.1 Hz, 2H), 4.48 (dd, *J* = 9.8, 4.3 Hz, 1H), 4.35 (q, *J* = 7.9 Hz, 1H), 3.05 (m, 1H), 2.89 (m, 3H), 2.42 (d, *J* = 1.5 Hz, 3H), 1.89 (s, 1H), 1.59 (s, 3H), 1.50 – 1.28 (m, 2H); ^{13}C NMR (101 MHz, CD_3OD+D_2O) δ 175.6, 174.6, 163.4, 162.7, 155.6, 155.4, 154.5, 144.1, 138.2, 130.4, 129.5, 129.5, 129.0, 128.6, 127.9, 126.8, 116.4, 115.9, 113.1, 106.4, 76.1, 67.6, 57.9, 56.6, 55.1, 40.5, 38.8, 30.2, 27.9, 23.3, 18.5; Purity >95% as determined by LCMS (5 to 95% MeCN in H_2O, R_t = 11.3/15 min); HRMS (ESI+) *m/z* calc. for $C_{42}H_{40}F_4N_4O_9H$ [M+H$^+$] = 821.28097, found 821.28219.

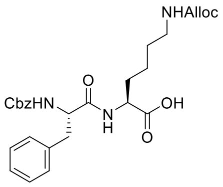

Z-FK(Alloc)-OH (14). Z-L-Phenylalanine (3.59 g, 12 mmol, 1.2 eq.) was treated with TSTU (3.61 g, 12 mmol, 1.2 eq.) and DiPEA (5.24 ml, 30 mmol, 3 eq.) in anhydrous DMF (25 ml) for 1 h at 0 °C. Full conversion to the OSu-ester was confirmed on TLC and H-L-Lys(alloc)-OH (2.30 g, 10 mmol, 1 eq.) was added. The resulting suspension was stirred for 16 h at r.t. leading to a clear solution. The reaction was poured into 1 N HCl and was extracted with EtOAc (3x). The extracts were combined, washed, dried and concentrated. Column chromatography (10 to 60% heptane in EtOAc + 1% AcOH) yielded a white solid (4.70 g, 9.19 mmol, 92%). R_f = 0.7 (40% EtOAc in heptane + 1% AcOH); ^{1}H NMR (500 MHz, MeOD) δ 7.34 – 7.16 (m, 11H), 6.00 – 5.86 (m, 1H), 5.28 (d, J = 17.3 Hz, 1H), 5.17 (d, J = 9.3 Hz, 1H), 5.03 (dd, J = 17.2, 11.8 Hz, 2H), 4.51 (d, J = 5.6 Hz, 2H), 4.47 (dd, J = 9.6, 5.0 Hz, 1H), 4.44 – 4.40 (m, 1H), 3.17 (dd, J = 14.0, 5.0 Hz, 1H), 3.12 – 3.05 (m, 2H), 2.98 (s, 1H), 2.91 – 2.82 (m, 2H), 1.94 – 1.83 (m, 1H), 1.77 – 1.66 (m, 1H), 1.51 (hept, J = 6.4 Hz, 2H), 1.42 (q, J = 8.3 Hz, 2H); ^{13}C NMR (126 MHz, MeOD) δ 173.7, 172.8, 163.5, 157.4, 156.8, 137.1, 136.8, 133.2, 129.0, 128.0, 128.0, 127.5, 127.3, 126.3, 116.0, 66.2, 64.9, 56.3, 52.2, 52.1, 40.1, 37.7, 35.6, 31.0, 30.3, 29.0, 22.5.

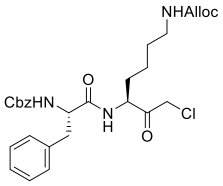

Z-FK(Alloc)-CMK (15). Z-FK(Alloc)-OH (771 mg, 1.51 mmol) was taken up in dry THF (5 ml), cooled to -15 °C and was treated with N-methyl morpholine (207 µl, 1.88 mmol, 1.25 eq.) and isobutyl chloroformate (235 µl, 1.81 mmol, 1.2 eq.) for 15 min at -15 °C. Diazomethane was prepared from Diazald (1.29 g, 6.03 mmol, 4 eq. in 30 ml Et_2O) by reaction with potassium hydroxide (1.0 g, 6.03 mmol, 4 eq. in 10 ml ethanol). The solution was distilled over a 65 °C water bath and the formed ethereal diazomethane was poured into the mixed anhydride. The reaction mixture was stirred for 3 hours at -15 °C before treating with concentrated HCl in AcOH (1:1, v:v, 6 ml). The reaction was poured into water and extracted with EtOAc (3x). The extracts were collected,

washed with sat. NaCl, dried over Na_2SO_4, filtered, and concentrated. Column chromatography (0 to 6% acetone in DCM) yielded the title compound as white powder (656 mg, 1.21 mmol, 80%). R_f = 0.6 (20% acetone in DCM); ^{1}H NMR (500 MHz, MeOD) δ 7.37 – 7.20 (m, 11H), 5.92 (ddt, J = 16.4, 10.7, 5.4 Hz, 1H), 5.30 (d, J = 17.8 Hz, 1H), 5.17 (d, J = 10.4 Hz, 1H), 5.07 (s, 2H), 4.51 (d, J = 4.5 Hz, 2H), 4.49 – 4.40 (m, 2H), 4.24 – 4.07 (m, 2H), 3.14 – 3.05 (m, 3H), 2.93 (dd, J = 13.7, 8.3 Hz, 1H), 1.90 – 1.81 (m, 1H), 1.59 (dd, J = 9.4, 4.7 Hz, 1H), 1.54 – 1.43 (m, 2H), 1.40 – 1.24 (m, 2H); ^{13}C NMR (126 MHz, MeOD) δ 208.7, 173.0, 157.0, 136.8, 133.1, 129.0, 128.2, 128.0, 127.6, 127.3, 126.6, 116.0, 105.0, 66.2, 64.9, 56.6, 56.4, 46.4, 39.9, 37.5, 29.3, 28.9, 22.4.

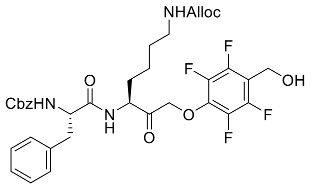

Z-FK(Alloc)-PMK (16). To a stirring solution of Z-FK(Alloc)-CMK (544 mg, 1 mmol) in dry DMF (10 ml) was added 2,3,5,6-tetrafluoro-4-(hydroxymethyl) phenol (216 mg, 1.1 mmol, 1.1 eq.) and potassium fluoride (87 mg, 1.5 mmol, 1.5 eq.). The reaction was stirred for 3 hours at 60 °C before pouring into sat. NH_4Cl and extraction with EtOAc (3x). The extracts were combined, washed with sat. NaCl, dried over Na_2SO_4, filtered, and concentrated. Column chromatography (0 to 8% acetone in DCM) yielded white powder (557 mg, 0.79 mmol, 79%). R_f = 0.3 (10% acetone in DCM); ^{1}H NMR (500 MHz, Acetone) δ 7.83 (s, 1H), 7.64 (d, J = 7.6 Hz, 1H), 7.22 – 7.02 (m, 10H), 6.52 (d, J = 8.3 Hz, 1H), 6.15 (s, 1H), 5.77 (dddd, J = 22.6, 16.2, 10.4, 5.3 Hz, 1H), 5.11 (dd, J = 17.1, 1.2 Hz, 1H), 5.03 – 4.96 (m, 2H), 4.94 – 4.85 (m, 3H), 4.56 (d, J = 1.8 Hz, 2H), 4.53 (t, J = 1.8 Hz, 2H), 4.45 – 4.39 (m, 1H), 4.38 – 4.32 (m, 3H), 3.06 (dd, J = 13.9, 5.8 Hz, 1H), 3.01 – 2.93 (m, 2H), 2.85 (dd, J = 14.2, 9.0 Hz, 1H), 1.82 – 1.71 (m, 1H), 1.55 – 1.45 (m, 1H), 1.42 – 1.29 (m, 2H), 1.29 – 1.17 (m, 2H); ^{13}C NMR (126 MHz, Acetone) δ 202.9, 171.8, 162.1, 156.2, 156.1, 146.4, 144.4, 141.19 (d, J = 16.0 Hz), 139.24 (d, J = 16.1 Hz), 137.6, 137.1, 133.9, 129.3, 128.3, 128.2, 127.7, 127.6, 126.5, 116.1, 113.06 (t, J = 18.6 Hz), 112.9, 75.1, 65.9, 64.5, 56.5, 55.8, 51.2, 40.2, 37.6, 35.4, 30.2, 22.4.

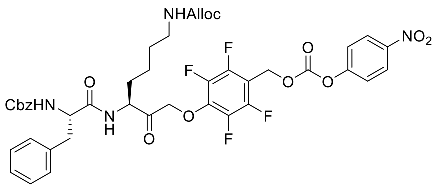

Z-Phe-Lys(Alloc)-PMK-PNP (18). To a stirring solution of 4-nitrophenylchloroformate (109 mg, 0.54 mmol, 1.5 eq.) and DMAP (132 mg, 1.08 mmol, 3 eq.) in anhydrous DCM at -10 °C was added alcohol **14** (254 mg, 0.36 mmol) as THF-solution dropwise over 15 min leading to a white suspension. The reaction was stirred for 3 h while warming to r.t. and was poured into sat. NH_4Cl. The aqueous phase was extracted with DCM (3x) and the extracts were washed with sat. NaCl, dried over Na_2SO_4, and concentrated. Column chromatography (0 to 10% acetone in DCM) yielded white solid (309 mg, 0.36 mmol, 99%). R_f = 0.8 (10% acetone in DCM); ^{1}H NMR (400 MHz, CDCl3) δ 8.31 – 8.25 (m, 2H), 7.42 – 7.37 (m, 2H), 7.36 – 7.15 (m, 13H), 6.68 (d, J = 7.5 Hz, 1H), 5.88 (ddt, J = 16.4, 10.8, 5.6 Hz, 1H), 5.44 (s, 1H), 5.39 (s, 2H), 5.32 – 5.14 (m, 2H), 5.08 (s, 2H), 5.03 – 4.95 (m, 1H), 4.89 (s, 2H), 4.73 – 4.63 (m, 1H), 4.53 (h, J = 3.1, 2.3 Hz, 2H), 4.49 – 4.44 (m, 1H), 3.18 (dd, J = 13.7, 6.8 Hz, 1H), 3.14 – 3.03 (m, 4H), 1.92 – 1.79 (m, 1H), 1.62 – 1.54 (m, 1H), 1.47 (tt, J = 14.1, 7.1 Hz, 2H); ^{13}C NMR (101 MHz, CDCl3) δ 202.5, 171.4, 156.7, 155.3, 152.0, 145.6, 138.9, 136.1, 132.9, 129.3, 128.7, 128.6, 128.3, 128.0, 127.2, 125.4, 121.7, 117.6, 75.2, 67.2, 65.6, 57.8, 56.2, 55.2, 39.9, 38.2, 29.8, 29.4, 21.9.

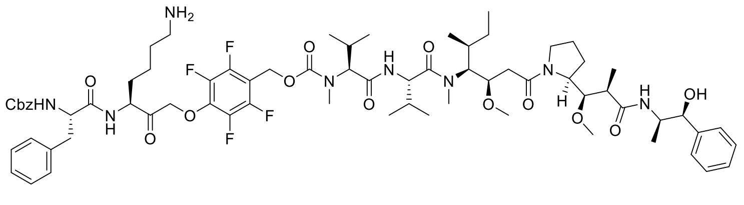

Z-FK-PMK-MMAE (2). To a solution of Z-FK(alloc)-PMK-PNP (13 mg, 15 µmol, 1.5 eq.) and MMAE (7.2 mg, 10 µmol, 1 eq.) in anhydrous DMF (1 ml) was added anhydrous HOBt-solution (0.5 M in DMF) (20 µl, 10 µmol, 1 eq.) and anhydrous pyridine (15 µl, 0.19 mmol, 19 eq.). The reaction was stirred for

Information Classification: General

48 h at r.t., before pouring into sat. NH_4Cl and extracting with EtOAc (3x). The extracts were combined, washed with sat. NaCl, dried over Na_2SO_4, filtered, and concentrated. The crude was taken up in anhydrous DCM (1 ml) and treated with palladium tetrakis triphenylphosphine (1.2 mg, 1 µmol, 0.1 eq.) and dimethyl barbituric acid (7 mg, 45 µmol, 4.5 eq.) for 15 min at r.t.. The mixture was diluted with DCM (3 ml) and purified by preparatory HPLC (5 to 65% MeCN in H_2O, R_t = 28/30 min). Subsequent lyophilisation (2x) yielded white powder (10.5 mg, 7.1 µmol, 71%). ^1H NMR (500 MHz, MeOD) δ 7.73 – 7.44 (m, 3H), 7.44 – 7.14 (m, 15H), 5.42 – 5.15 (m, 2H), 5.11 – 4.98 (m, 2H), 4.71 – 4.53 (m, 2H), 4.44 – 4.33 (m, 1H), 4.29 – 4.16 (m, 2H), 4.16 – 4.03 (m, 1H), 3.87 (d, *J* = 9.1 Hz, 1H), 3.75 – 3.65 (m, 1H), 3.60 – 3.53 (m, 1H), 3.47 – 3.40 (m, 1H), 3.38 – 3.35 (m, 3H), 3.28 (s, 1H), 3.25 – 3.18 (m, 1H), 3.11 (s, 1H), 3.10 – 2.93 (m, 3H), 2.92 – 2.79 (m, 4H), 2.58 – 2.45 (m, 2H), 2.30 – 2.13 (m, 2H), 2.12 – 1.99 (m, 1H), 1.99 – 1.88 (m, 2H), 1.87 – 1.76 (m, 2H), 1.75 – 1.65 (m, 1H), 1.65 – 1.50 (m, 3H), 1.52 – 1.34 (m, 3H), 1.23 – 1.12 (m, 6H), 1.13 – 1.06 (m, 1H), 1.04 – 0.92 (m, 6H), 0.92 – 0.79 (m, 9H); ^{13}C NMR (126 MHz, MeOD) δ 203.3, 199.1, 174.3, 174.0, 173.7, 173.4, 173.2, 170.4, 170.3, 165.1, 161.6, 156.9, 146.7, 144.8, 142.7, 142.4, 136.8, 134.5, 134.3, 131.9, 129.0, 128.9, 128.5, 128.2, 128.2, 128.1, 128.0, 127.8, 127.6, 127.3, 127.2, 127.2, 127.0, 126.6, 126.5, 85.3, 82.1, 78.2, 77.5, 76.1, 75.9, 74.7, 66.2, 64.7, 60.6, 60.1, 59.4, 59.2, 57.2, 57.0, 56.8, 56.5, 54.7, 50.0, 49.4, 46.7, 44.5, 44.1, 39.1, 39.0, 37.3, 35.4, 30.4, 29.3, 28.9, 28.5, 26.5, 26.3, 25.6, 25.2, 24.4, 24.2, 23.0, 22.4, 21.9, 18.3, 17.6, 14.4, 13.7, 9.4; Purity >95% as determined by LCMS (5 to 100% MeCN in H_2O, R_t = 6.6/10 min); HRMS (ESI+) calc. for $C_{71}H_{98}F_4N_8O_{14}$ $[M+H]^+$ = 1363.72168, found 1363.71852.

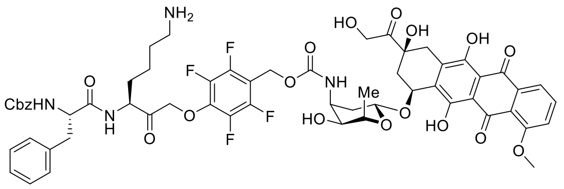

Z-FK-PMK-DOX (3). To a stirring solution of Z-FK(alloc)-PMK-PNP (39.1 mg, 45 µmol, 1.5 eq.) and Doxorubicin hydrochloride (19.4 mg, 30 µmol, 1 eq.) in anhydrous DMF (1 ml) was added DiPEA (27 µl, 150 µmol, 5 eq.). The reaction was stirred for 16 h at r.t., before pouring into sat. NH_4Cl and extracting with EtOAc (3x). The extracts were combined, washed with sat. NaCl, dried over Na_2SO_4, filtered, and concentrated. The crude was taken up in anhydrous DCM (1 ml), purged with argon for 10 min and treated with palladium tetrakis triphenylphosphine (3.5 mg, 3 µmol, 0.1 eq.) and 1,3-dimethylbarbituric acid (14.4 mg, 90 µmol, 3 eq.). The mixture was stirred for 5 min at r.t., diluted with MeOH and purified by preparatory HPLC (0 to 50% MeCN in H_2O, R_t = 29/30 min). Subsequent lyophilisation (2x) yielded orange powder (23.0 mg, 17.4 µmol, 59%). ^1H NMR (500 MHz, DMSO-d6) δ 14.00 (s, 1H), 13.24 (s, 1H), 8.55 (t, *J* = 7.5 Hz, 1H), 7.90 – 7.86 (m, 2H), 7.84 – 7.75 (m, 3H), 7.68 – 7.59 (m, 2H), 7.35 – 7.13 (m, 11H), 6.97 (dd, *J* = 7.9, 3.9 Hz, 1H), 5.44 (s, 1H), 5.28 – 5.13 (m, 2H), 5.13 – 4.94 (m, 4H), 4.92 (s, 2H), 4.58 (s, 2H), 4.35 – 4.20 (m, 2H), 4.15 (q, *J* = 6.6 Hz, 1H), 3.97 (s, 3H), 3.68 (dq, *J* = 11.5, 4.4, 3.5 Hz, 1H), 3.46 – 3.41 (m, 1H), 3.04 – 2.87 (m, 3H), 2.85 – 2.77 (m, 1H), 2.77 – 2.65 (m, 2H), 2.23 – 2.06 (m, 2H), 1.83 (td, *J* = 12.9, 3.8 Hz, 1H), 1.69 (dddt, *J* = 51.3, 13.8, 10.3, 5.3 Hz, 1H), 1.57 – 1.41 (m, 4H), 1.37 – 1.21 (m, 1H), 1.12 (d, *J* = 6.4 Hz, 3H); ^{13}C NMR (126 MHz, DMSO-d6) δ 214.2, 204.2, 203.8, 186.9, 186.8, 172.8, 172.6, 161.2, 158.6, 158.3, 156.5, 156.4, 156.4, 155.0, 155.0, 146.5, 144.5, 141.0, 139.1, 138.2, 138.1, 137.4, 137.3, 136.6, 136.0, 135.1, 134.5, 129.7, 129.7, 128.7, 128.7, 128.5, 128.5, 128.2, 128.2, 128.0, 128.0, 126.8, 126.8, 120.4, 120.2, 119.4, 111.2, 111.1, 108.7, 100.7, 75.4, 75.3, 70.3, 68.3, 67.1, 65.8, 64.2, 57.0, 56.8, 56.6, 55.9, 55.8, 53.3, 47.8, 39.0, 38.9, 37.7, 37.5, 37.0, 32.5, 30.1, 28.9, 26.9, 26.9, 22.4, 22.3, 17.5; Purity >95% as determined by LCMS (5 to 100% MeCN in H_2O, R_t = 5.9/10 min); HRMS (ESI+) calc. for$C_{59}H_{60}F_4N_4O_{18}$ $[M+H]^+$ = 1189.39170, found 1189.39012.

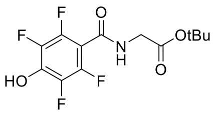

tert-butyl (2,3,5,6-tetrafluoro-4-hydroxybenzoyl)glycinate (21). To a stirring solution of 2,3,5,6-tetrafluoro-4-hydroxybenzoic acid (1.05 g, 5 mmol, 1 eq.) and H-L-Gly-OtBu (0.92 g, 5.5 mmol, 1.1 eq.) in dry DMF (10 ml) was added EDCI (0.85 g, 5.5 mmol, 1.1 eq.), HOBt

(0.84 g, 5.5 mmol, 1.1 eq.) and NMM (0.6 ml, 5.5 mmol, 1.1 eq.). The reaction mixture was stirred 16 h at r.t., before pouring into sat. NH_4Cl and extracting with DCM (3x). The extracts were washed with sat. Nacl, dried over Na_2SO_4, filtered, and concentrated. Flash column chromatography (20 to 30% EtOAc in PetEt) yielded white solid (1.45 g, 4.5 mmol, 90%). R_f = 0.4 (60% EtOAc in PetEt); ^{1}H NMR (500 MHz, Acetone) δ 7.45 (t, *J* = 6.2 Hz, 1H), 4.09 (d, *J* = 5.9 Hz, 2H), 1.59 (s, 9H); ^{13}C NMR (126 MHz, Acetone) δ 168.3, 158.9, 145.4, 143.4, 139.1, 137.7, 137.2, 117.4, 106.2, 81.6, 42.3, 27.3.

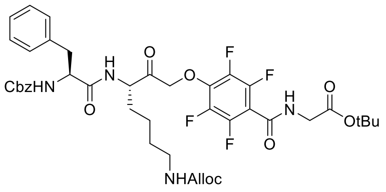

Z-FK(Alloc)-PMK-G-tBu (22). To a stirring solution of Z-FK(Alloc)-CMK (109 mg, 0.2 mmol, 1 eq.) was added phenol **21** (71 mg, 0.22 mmol, 1.1 eq.) and potassium fluoride (17.4 mg, 0.3 mmol, 1.5 eq.). The solution was stirred for 16 hours at 60 °C, before pouring into sat. NH_4Cl and extracting with EtOAc (3x). The extracts were washed with sat. NaCl, dried over Na_2SO_4, filtered, and concentrated. Column chromatography (0 to 30% EtOAc in PetEt) yielded the title compound as white solid (150 mg, 0.18 mmol, 90%). R_f = 0.8 (80% EtOAc in PetEt); ^{1}H NMR (500 MHz, DMSO-d6) δ 9.22 (t, *J* = 5.9 Hz, 1H), 8.49 (d, *J* = 7.4 Hz, 1H), 7.64 (q, *J* = 8.3 Hz, 1H), 7.38 – 7.13 (m, 11H), 5.89 (ddt, *J* = 17.1, 10.5, 5.3 Hz, 1H), 5.26 (dq, *J* = 17.3, 1.8 Hz, 1H), 5.19 – 5.13 (m, 2H), 5.08 – 5.02 (m, 1H), 4.97 (s, 2H), 4.45 (d, *J* = 5.4 Hz, 2H), 4.31 (td, *J* = 8.0, 6.6, 4.0 Hz, 2H), 3.98 – 3.88 (m, 2H), 3.04 – 2.90 (m, 3H), 2.87 – 2.75 (m, 1H), 1.76 (td, *J* = 9.6, 8.9, 5.2 Hz, 1H), 1.59 – 1.46 (m, 1H), 1.44 (s, 9H), 1.44 – 1.33 (m, 2H), 1.33 – 1.17 (m, 2H); ^{13}C NMR (126 MHz, DMSO-d6) δ 203.9, 172.5, 168.4, 158.2, 156.4, 144.7, 142.7, 140.9, 139.0, 138.3, 137.8, 137.4, 134.3, 129.7, 128.7, 128.5, 128.2, 128.0, 126.8, 117.3, 110.2, 81.4, 75.3, 65.8, 64.6, 56.5, 56.0, 42.5, 37.6, 29.5, 29.2, 28.1, 22.8.

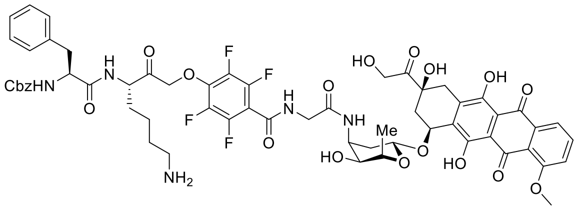

Z-FK-PMK-G-Dox (23). Z-FK(Alloc)-PMK-G-OtBu (39 mg, 47 µmol, 1.1 eq.) was taken up in DCM (1 ml) and treated with TFA (1 ml) for 2.5 h at r.t., before co-evarporation with toluene (3x). The crude was taken up in dry DMF (1 ml) and Doxorubicin hydrochloride (25 mg, 43 µmol, 1 eq.), EDCI (7.4 mg, 52 µmol, 1.2 eq.), HOBt (7.9 mg, 52 µmol, 1.2 eq.) and a drop of DiPEA were added. The reaction was stirred 16 h at r.t. in the dark, after which the reaction was poured into water. The aqueous layer was extracted with EtOAc (3x) and the extracts were washed with sat. NaCl, dried over Na_2SO_4, filtered, and concentrated. The crude was taken up in DCM (1 ml), purged with argon for 10 min and palladium tetrakis triphenylphosphine (5 mg, 4.3 mmol, 0.1 eq.) and dimethylbarbituric acid (20 mg, 129 mmol, 3 eq.) were added. The reaction was stirred for 5 min at r.t. and full conversion was confirmed via LCMS. The mixture was diluted with methanol and the mixture was purified by prep HPLC (0 to 40% MeCN in H_2O, 29/30 min). Subsequent lyophilisation (2x) yielded orange powder (30 mg, 23 µmol, 52%). ^{1}H NMR (500 MHz, DMSO-d6) δ 14.01 (s, 1H), 13.25 (s, 1H), 9.00 (t, *J* = 5.8 Hz, 1H), 8.47 (d, *J* = 7.5 Hz, 1H), 7.91 – 7.84 (m, 2H), 7.66 (d, *J* = 8.1 Hz, 1H), 7.65 – 7.60 (m, 2H), 7.36 – 7.22 (m, 10H), 7.22 – 7.13 (m, 2H), 5.88 (ddt, *J* = 17.3, 10.5, 5.3 Hz, 1H), 5.45 (s, 1H), 5.30 – 5.21 (m, 2H), 5.17 – 5.09 (m, 2H), 5.07 – 4.98 (m, 2H), 4.95 (s, 2H), 4.93 (d, *J* = 4.0 Hz, 1H), 4.59 (s, 2H), 4.46 – 4.42 (m, 2H), 4.29 (td, *J* = 9.4, 5.4 Hz, 2H), 4.19 (q, *J* = 6.7 Hz, 1H), 4.06 – 3.99 (m, 1H), 3.97 (s, 3H), 3.94 – 3.82 (m, 2H), 3.42 (s, 1H), 3.05 – 2.87 (m, 5H), 2.78 (dd, *J* = 13.8, 9.9 Hz, 1H), 2.25 – 2.19 (m, 1H), 2.16 – 2.07 (m, 1H), 1.85 (td, *J* = 12.9, 3.9 Hz, 1H), 1.74 (ddt, *J* = 13.9, 10.1, 5.3 Hz, 1H), 1.56 – 1.45 (m, 2H), 1.44 – 1.33 (m, 2H), 1.31 – 1.18 (m, 2H), 1.15 (d, *J* = 6.5 Hz, 3H); ^{13}C NMR (126 MHz, DMSO-d6) δ 214.2, 203.8, 187.0, 186.9, 172.5, 167.3, 161.2, 157.9, 156.6, 156.3, 154.9, 144.6, 142.7, 140.9, 138.9, 138.3, 137.4, 136.6, 135.9, 135.1, 134.5, 134.3, 129.7, 128.7, 128.5, 128.2, 128.0, 126.8, 120.5, 120.2, 119.4, 117.3, 111.2, 111.1, 110.5, 105.0,

100.8, 75.4, 75.3, 70.5, 68.4, 67.1, 65.8, 64.6, 64.2, 57.0, 56.5, 56.0, 45.6, 42.9, 37.6, 37.1, 32.5, 30.2, 29.5, 29.2, 22.8, 17.5; Purity >95% as determined by LCMS (5 to 100% MeCN in H_2O, R_t = 6.1/10 min); HRMS (ESI+) calc. for $C_{60}H_{61}F_4N_5O_{18}$ $[M+H]^+$ = 1216.40260, found 1216.40123.

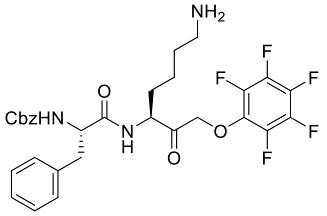

Z-FK-PMK (FJD005). To a stirring solution of Z-FK(Boc)-CMK (30.0 mg, 53.6 µmol, 1 eq.) was added pentafluorophenol (11.8 mg, 64.2 µmol, 1.2 eq.) and potassium fluoride (4.7 mg, 80 µmol, 1.5 eq.). The reaction was stirred for 2 h at 80 °C. The mixture was poured into water, extracted with EtOAc (3x) and the extracts were washed with sat. NaCl, dried over Na_2SO_4, filtered, and concentrated. The crude was taken up in DCM (1 ml) and was treated with TFA (1 ml) at r.t. for 30 min, before co-evaporation with toluene (3x). The crude was dissolved in MeOH and was purified by prep HPLC (5 to 60% MeCN in H_2O, 26/30 min). Subsequent lyophilisation (2x) yielded the title compound as white powder (22 mg, 36 µmol, 68%). ^{1}H NMR (500 MHz, MeOD) δ 7.25 – 7.12 (m, 10H), 7.07 (tq, *J* = 5.5, 2.8, 2.4 Hz, 1H), 4.99 – 4.87 (m, 2H), 4.66 (q, *J* = 17.6 Hz, 1H), 4.39 (dd, *J* = 9.9, 4.3 Hz, 1H), 4.28 (t, *J* = 7.7 Hz, 1H), 3.03 – 2.81 (m, 2H), 2.76 (t, *J* = 7.7 Hz, 2H), 1.86 – 1.76 (m, 1H), 1.60 – 1.41 (m, 3H), 1.38 – 1.24 (m, 2H); ^{13}C NMR (126 MHz, MeOD) δ 202.7, 173.2, 156.9, 142.0, 140.1, 138.0, 137.0, 136.8, 129.0, 128.1, 128.1, 127.6, 127.2, 126.5, 75.1, 66.2, 56.5, 55.3, 39.1, 37.4, 28.7, 26.5, 22.0; Purity >95% as determined by LCMS (5 to 100% MeCN in H_2O, R_t = 18.7/30 min); HRMS (ESI+) calc. for $C_{30}H_{61}F_5N_3O_5$ $[M+H]^+$ = 608.21839, found 608.21766.

Information Classification: General

NMR spectra

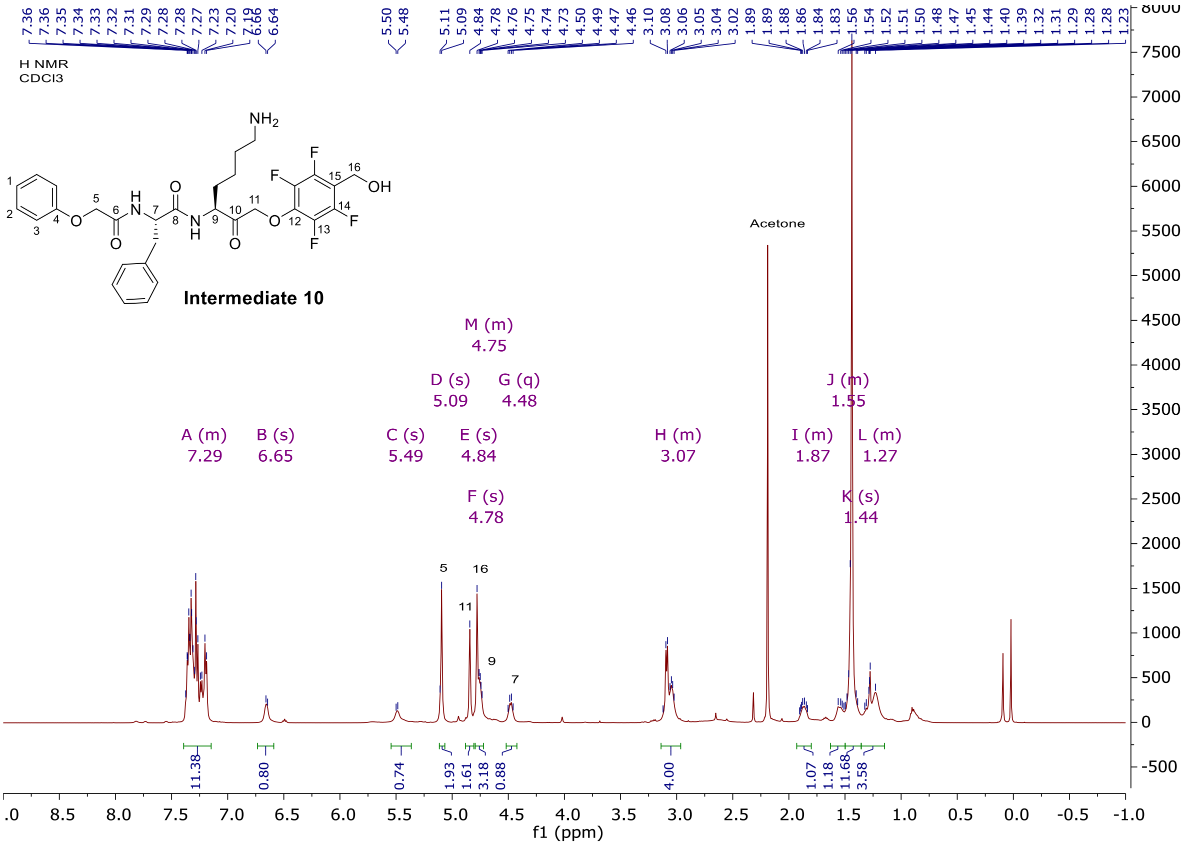
H NMR
CDCl3
Intermediate 10
Acetone
A (m) 7.29
B (s) 6.65
C (s) 5.49
D (s) 5.09
E (s) 4.84
F (s) 4.78
G (q) 4.48
H (m) 3.07
I (m) 1.87
J (m) 1.55
K (s) 1.44
L (m) 1.27
M (m) 4.75
f1 (ppm)

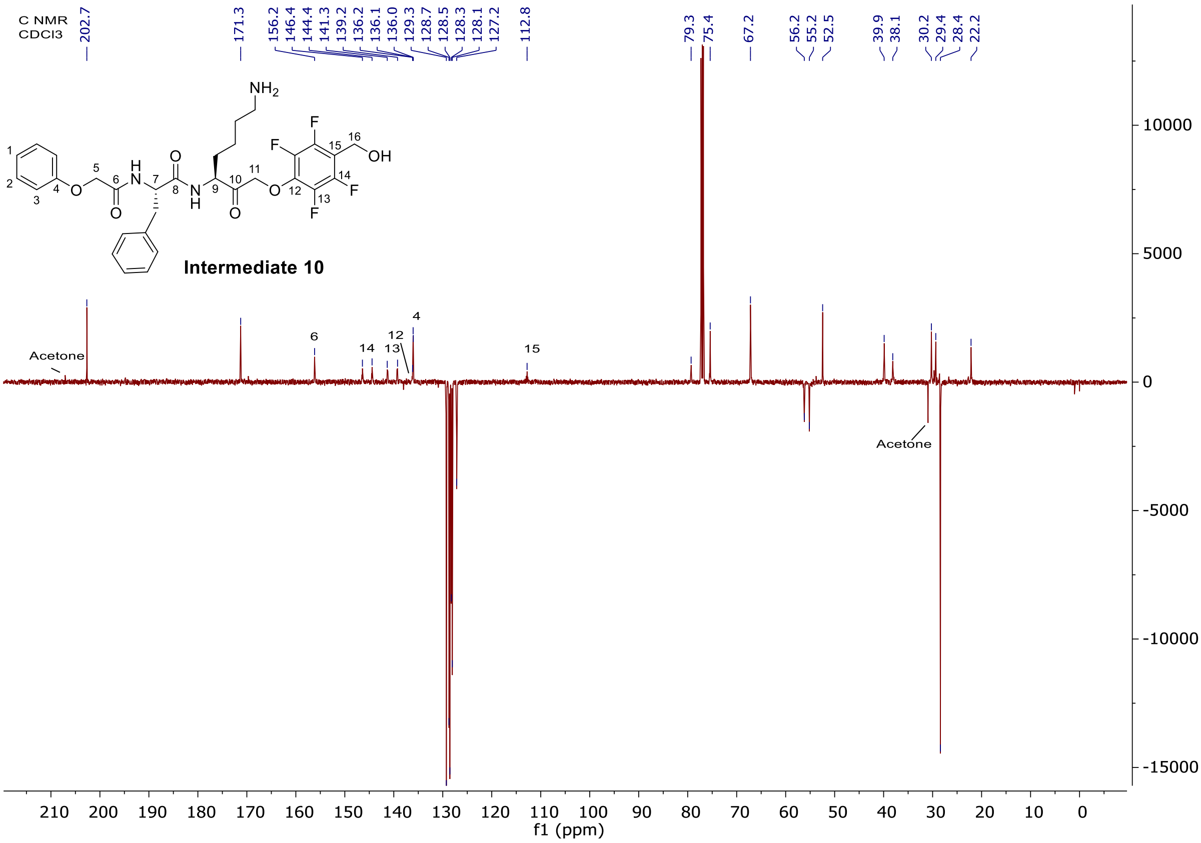
C NMR
CDCl3
Intermediate 10
Acetone
Acetone
f1 (ppm)

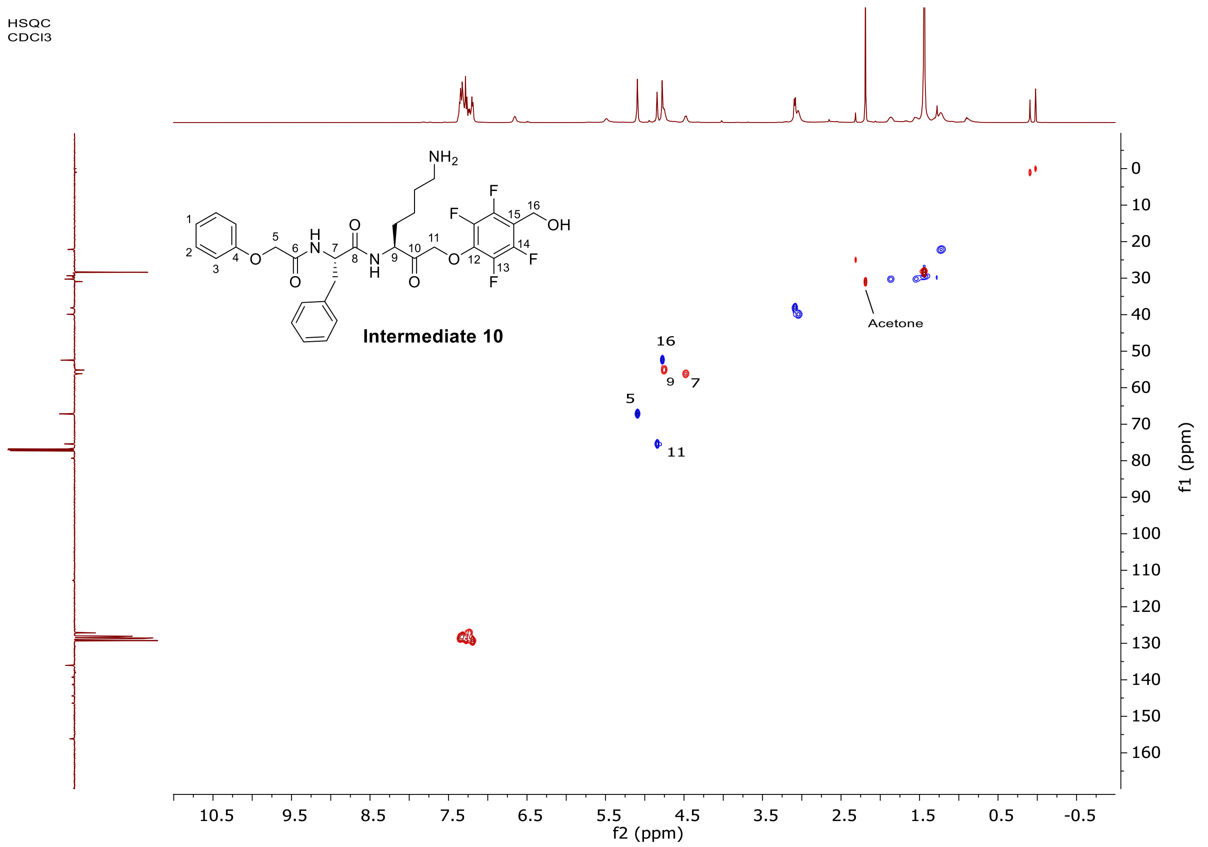

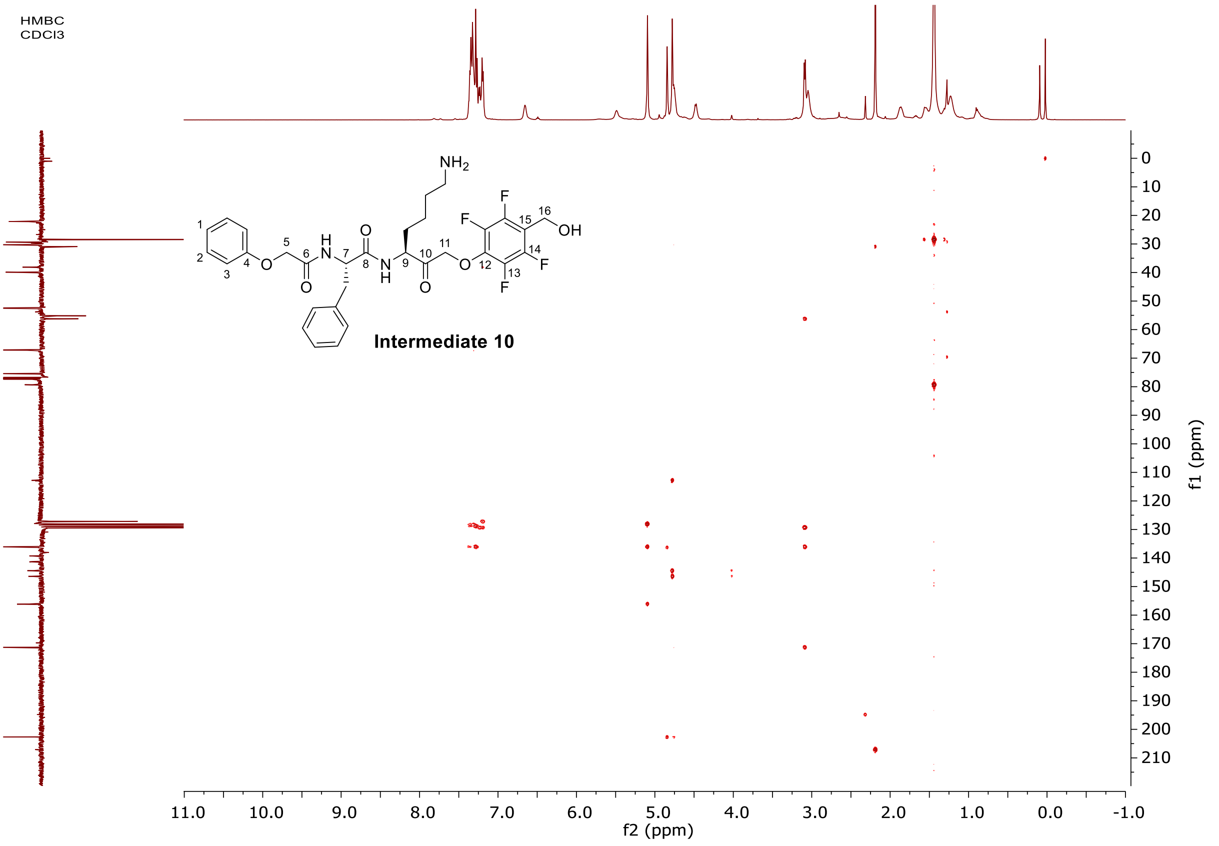

Information Classification: General

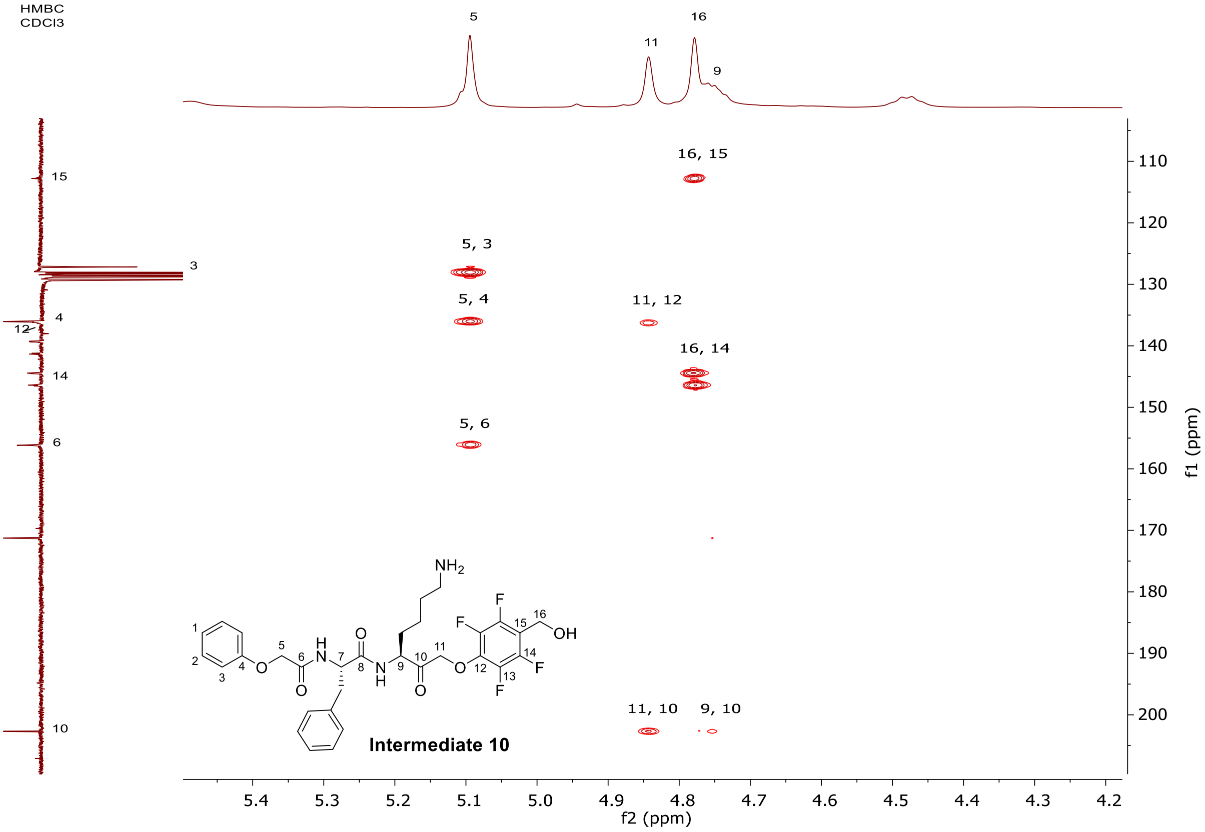

For clarity only the region of interest is annotated. HMBC interactions are indicated with the location of the proton followed by the interacting carbon atom. The interactions between the protons at location 11 and carbon atoms at location 10 and 12, confounded by the lack of interaction between the protons at location 11 and the carbon atom at location 16, show that the presented orientation of the phenoxymethyl ketone is correct.

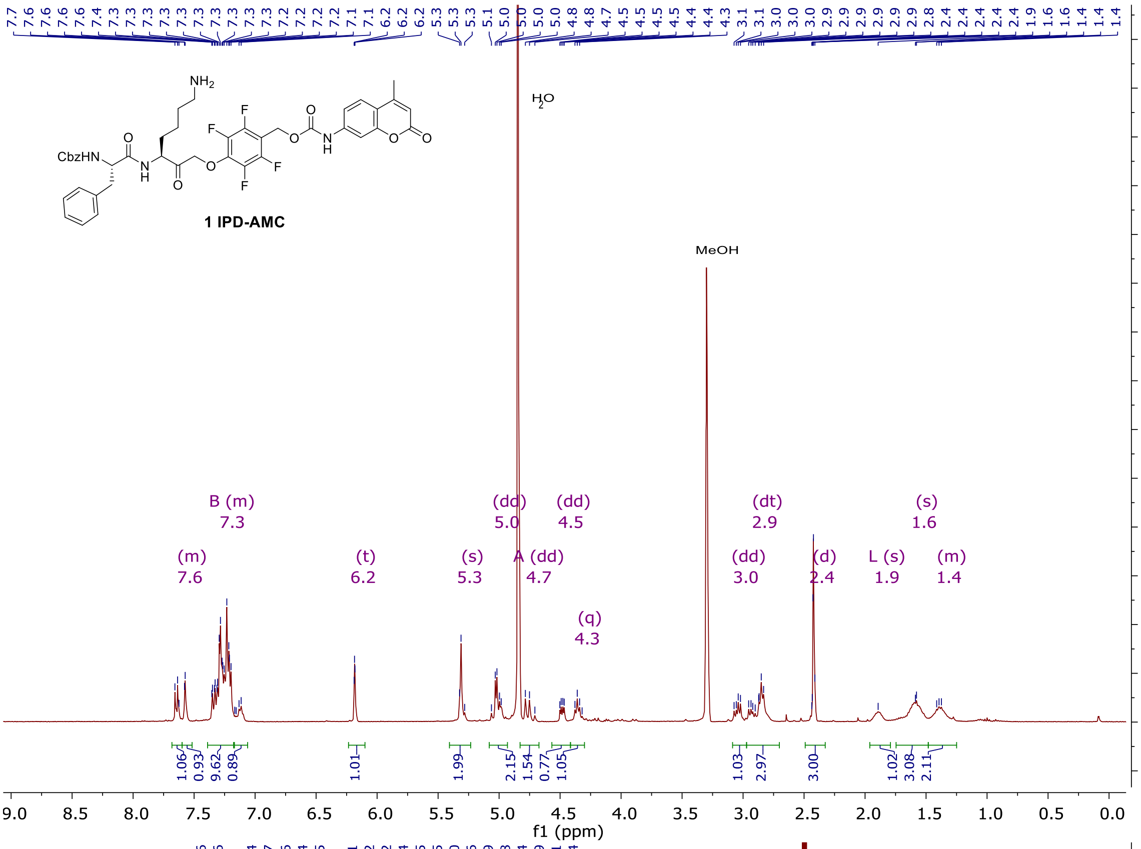

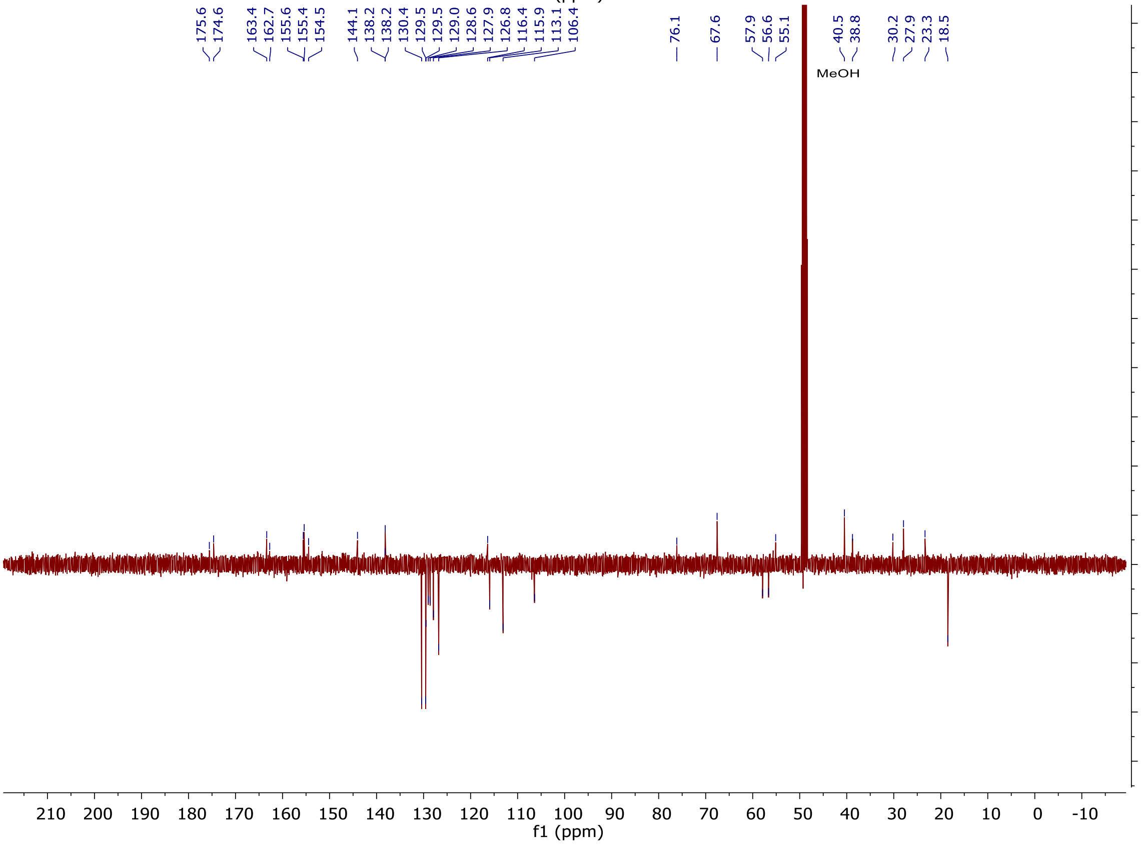

Information Classification: General

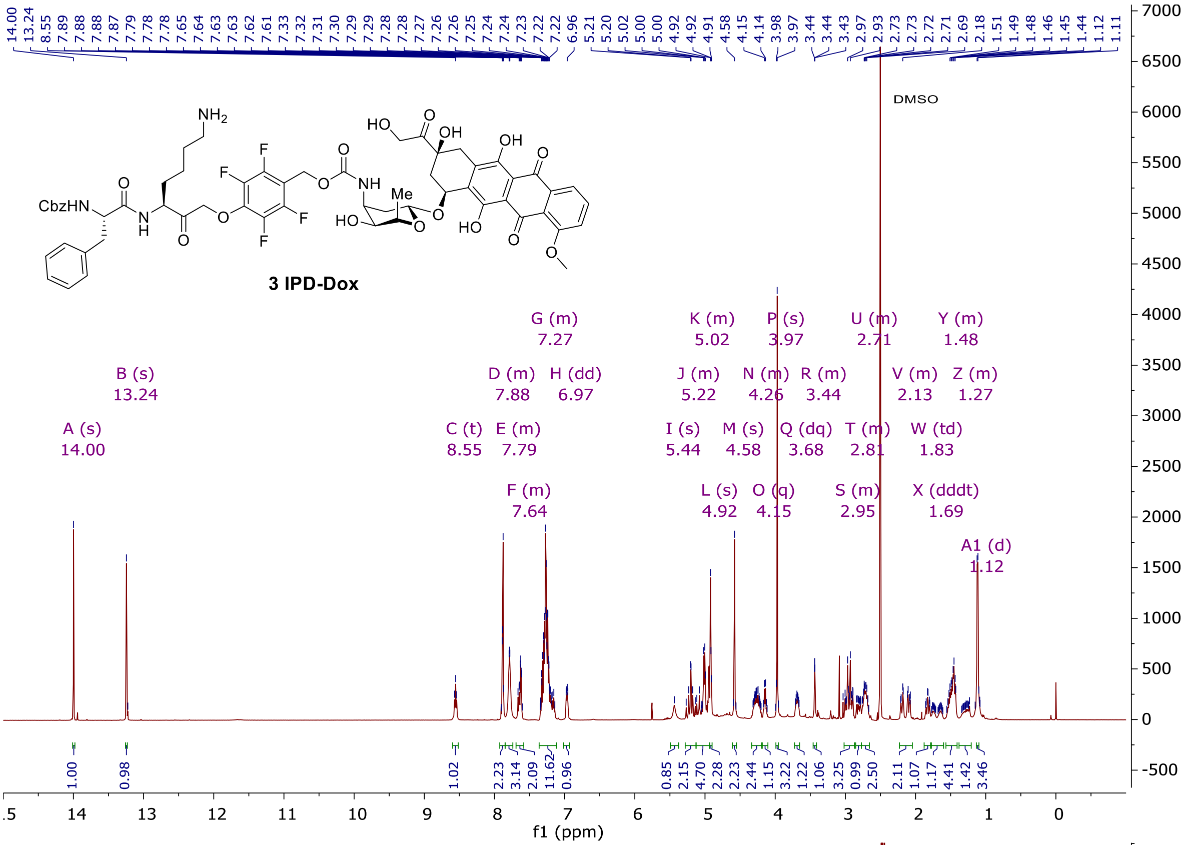

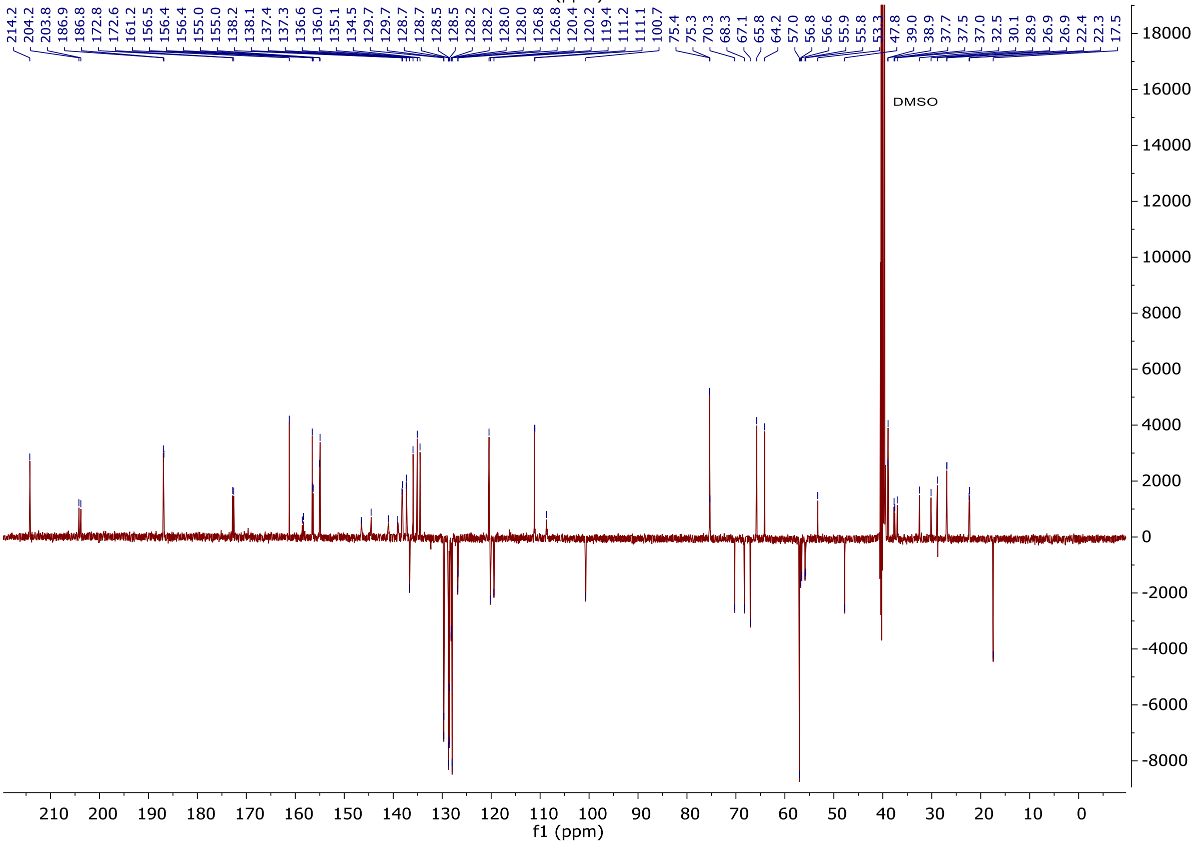

Information Classification: General

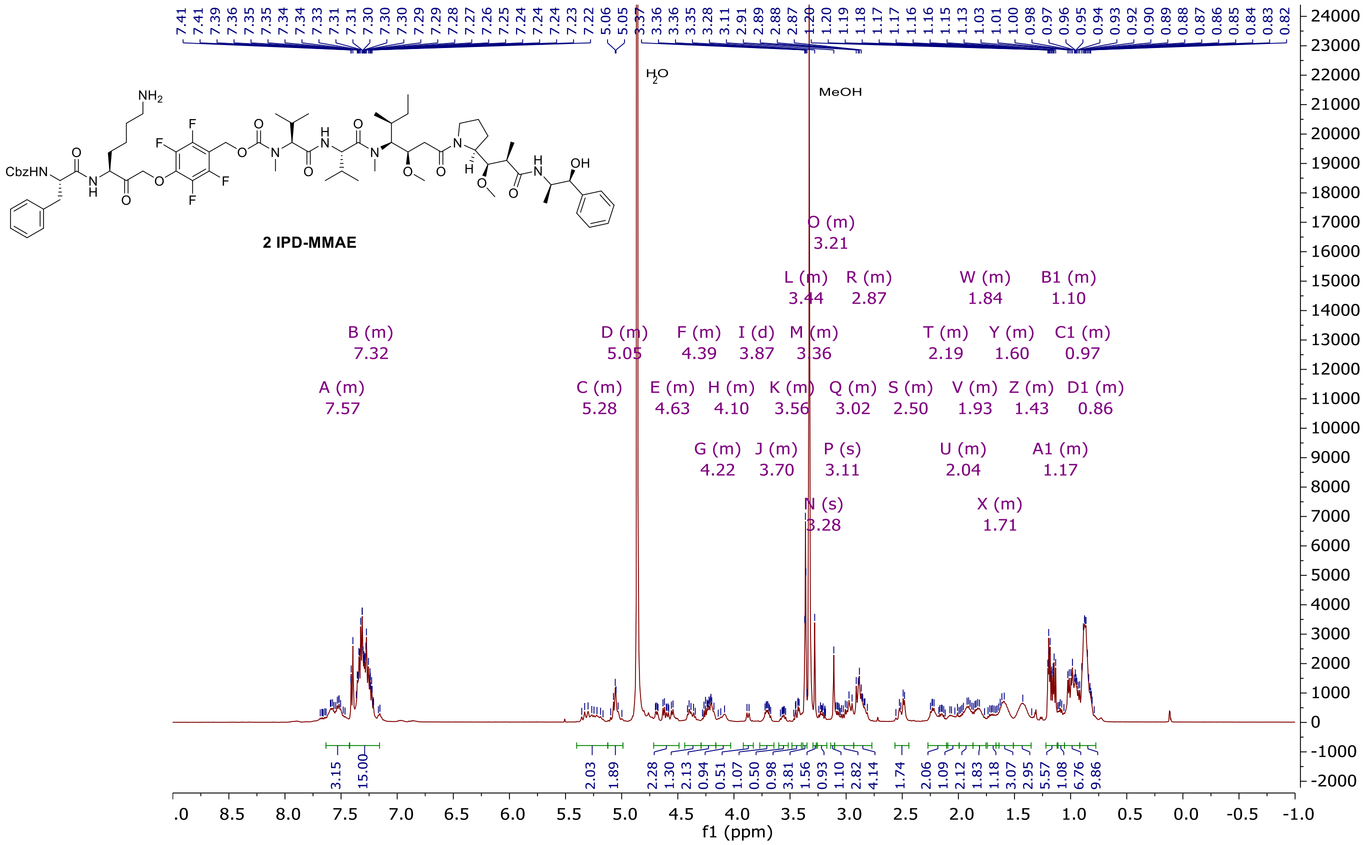

2 IPD-MMAE

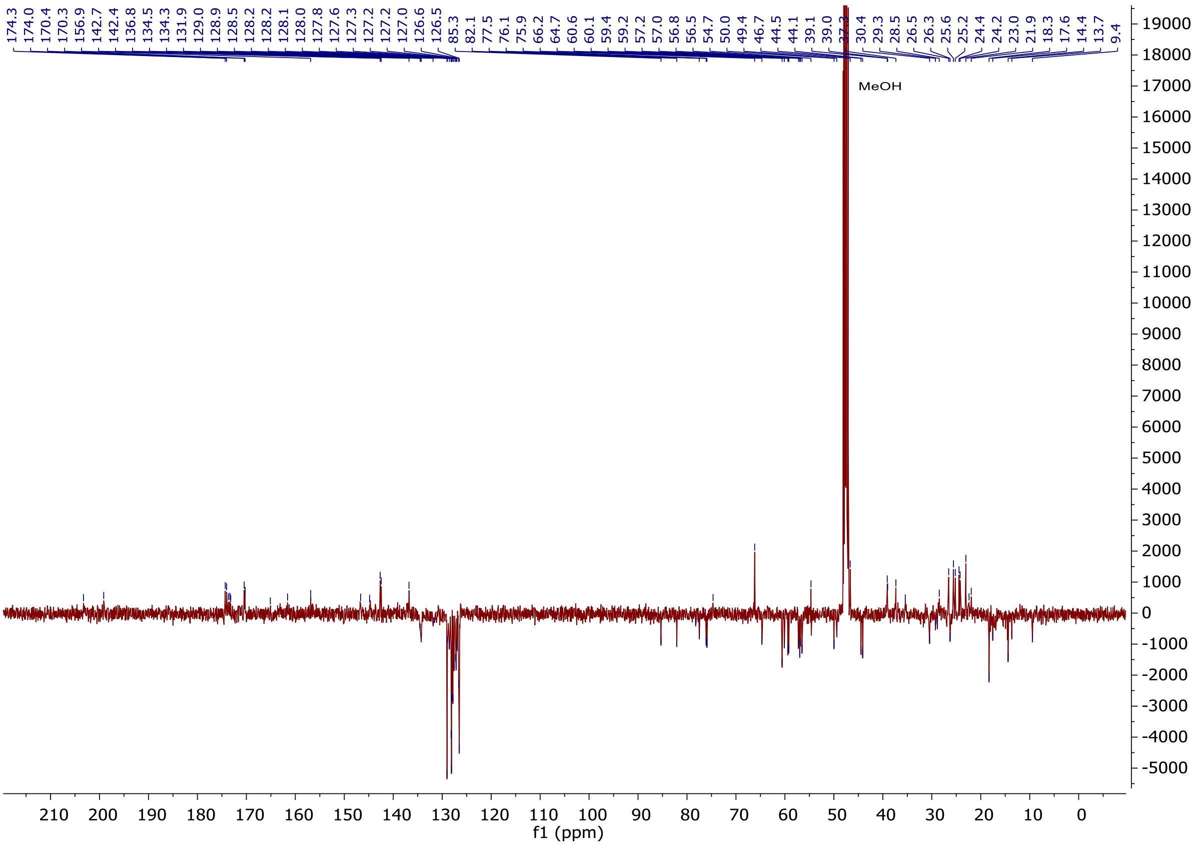

Information Classification: General

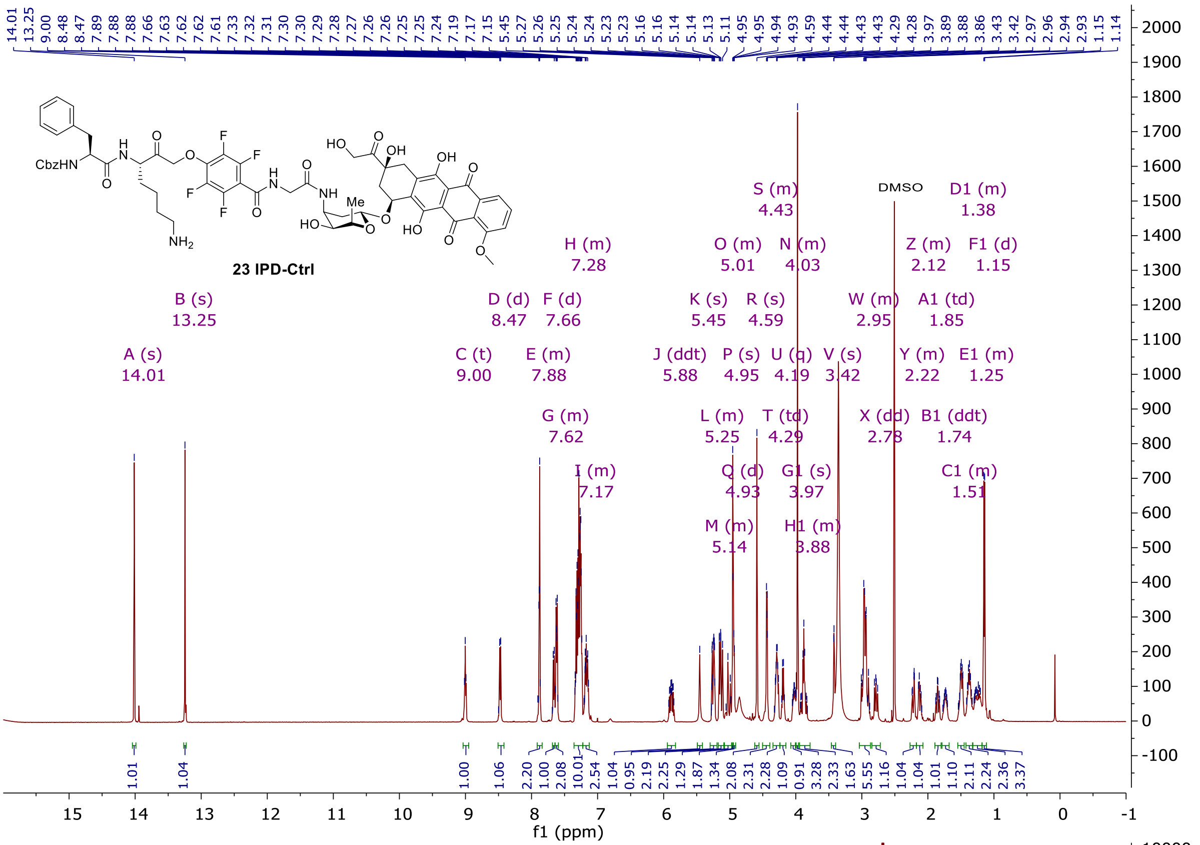

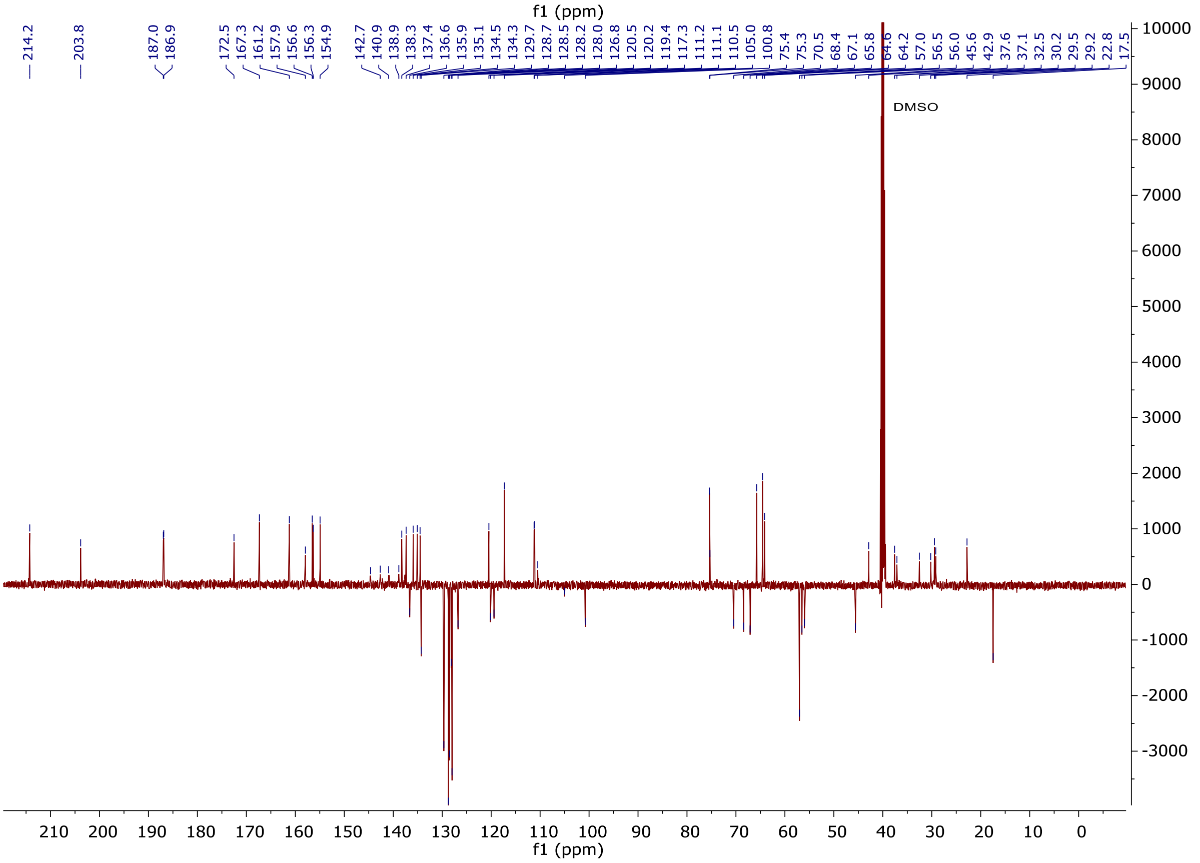

Information Classification: General

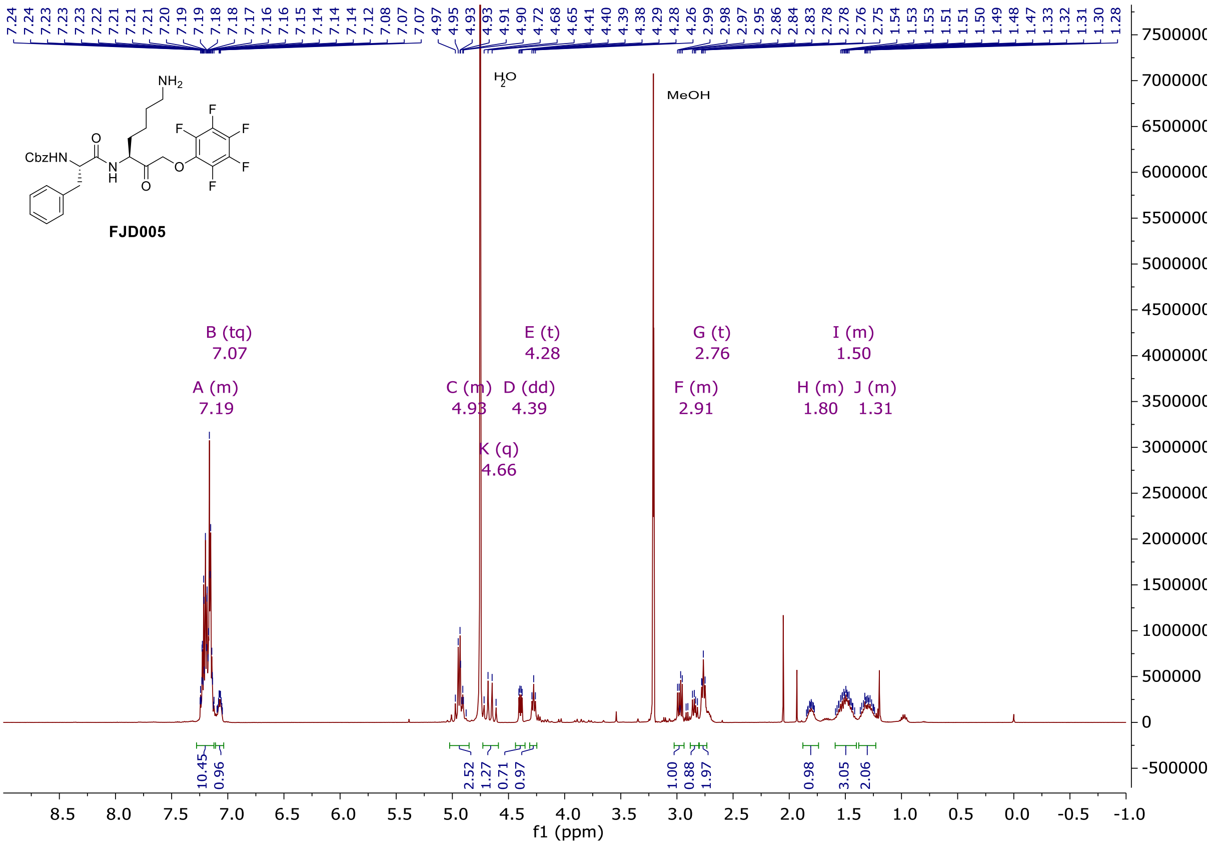
FJD005
H2O
MeOH
A (m) 7.19
B (tq) 7.07
C (m) 4.93
D (dd) 4.39
E (t) 4.28
K (q) 4.66
F (m) 2.91
G (t) 2.76
H (m) 1.80
I (m) 1.50
J (m) 1.31
f1 (ppm)

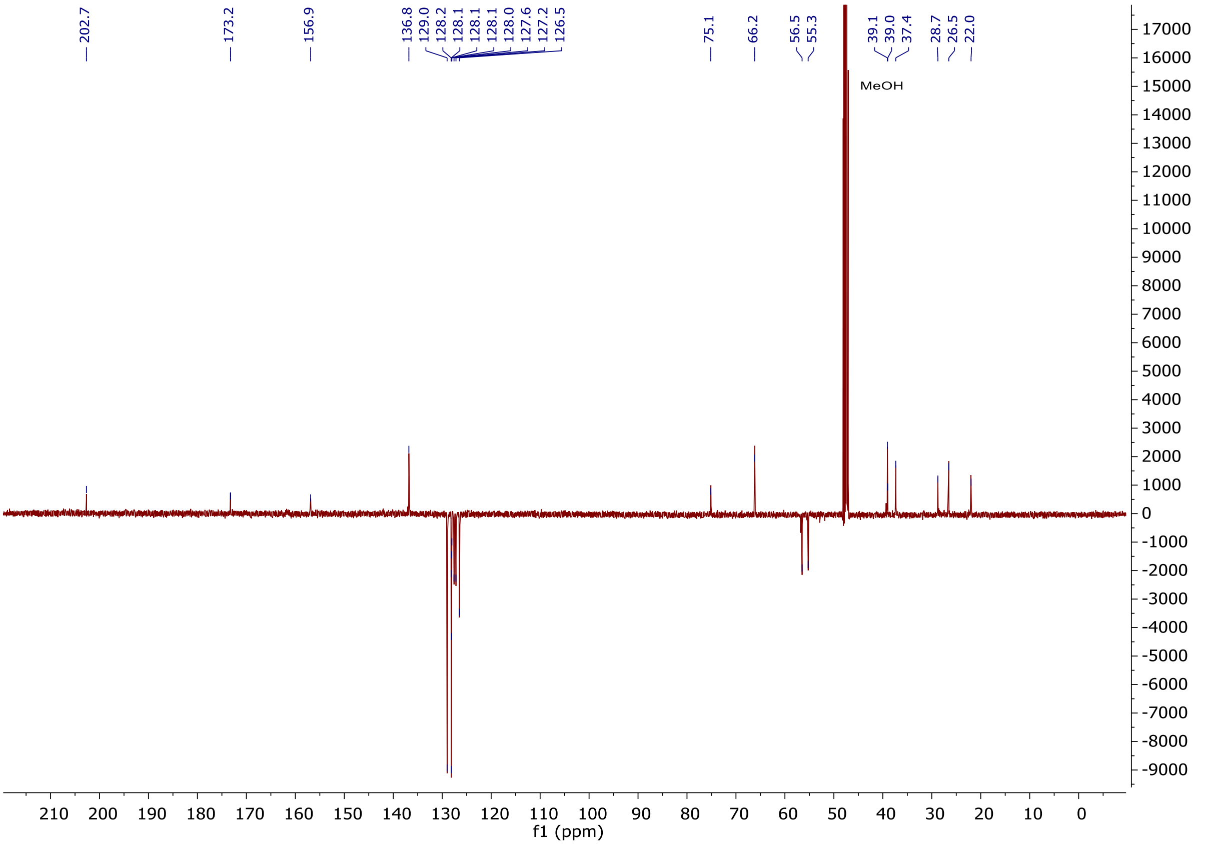
202.7
173.2
156.9
136.8
129.0
128.2
128.1
128.1
128.1
128.0
127.6
127.2
126.5
75.1
66.2
56.5
55.3
39.1
39.0
37.4
28.7
26.5
22.0
MeOH
f1 (ppm)
